# Metaverse-Based Virtual Reality for Remote Anatomy Education: Pilot Randomized Controlled Trial

**DOI:** 10.2196/93092

**Published:** 2026-05-19

**Authors:** Jason Ha, Andrew Gan, Ameen Mahmood, Ahmed Salih, Princewill Ukpeh, Clement Yong, Adharsh Prasad, Arushi Rastogi, Ellen Martin, Swati Mulchandani, Keshav Krishnan, Nicole Salgado Fernandez, Krish Gupta, Florence Chang, Srija Gullapalli, Leena Abdalla, Mazen Kafienah, Jagtar Dhanda

**Affiliations:** 1 Bristol Medical School, University of Bristol, First Floor, 5 Tyndall Avenue, Bristol, England, BS8 1UD, United Kingdom, 44 7904492270; 2 Birmingham Medical School, University of Birmingham, Birmingham, England, United Kingdom; 3 Faculty of Medicine, Imperial College London, London, England, United Kingdom; 4 UCL Medical School, University College London, London, England, United Kingdom; 5 Edinburgh Medical School, University of Edinburgh, Edinburgh, Scotland, United Kingdom; 6 School of Medicine, Dentistry and Biomedical Sciences, Queen's University Belfast, Belfast, Northern Ireland, United Kingdom; 7 Leeds School of Medicine, University of Leeds, Leeds, England, United Kingdom; 8 Buckingham Medicine School, University of Buckingham, Buckingham, England, United Kingdom; 9 School of Medicine, Cardiff University, Cardiff, Wales, United Kingdom; 10 Barts and The London School of Medicine and Dentistry, Queen Mary University of London, London, England, United Kingdom; 11 GKT School of Medical Education, King's College London, London, England, United Kingdom; 12 Head and Neck Oncology, Queen Victoria Hospital, East Grinstead, England, United Kingdom; 13 Brighton and Sussex Medical School, Brighton, England, United Kingdom

**Keywords:** virtual reality, extended reality, metaverse, anatomy education, medical education, tracheostomy, remote learning

## Abstract

**Background:**

Traditional anatomy teaching relies on cadaveric dissection and 2D resources, which often require in-person attendance and may limit spatial understanding. Virtual reality (VR) provides an immersive, remote alternative that supports 3D visualization from home. Recent evidence suggests that while VR may yield comparable factual knowledge gains to 2D methods, its primary value lies in enhancing learner engagement, motivation, and perceived educational value.

**Objective:**

This pilot randomized controlled trial compares remote synchronized VR with didactic animated anatomy lectures for the teaching of tracheostomy anatomy.

**Methods:**

Participants were recruited via convenience sampling through the VRiMS (Virtual Reality in Medicine and Surgery) Surgical Society network. All participants first attended a synchronous 20-minute online lecture delivered by a consultant surgeon. They were then individually randomized to one of 2 groups using a computer-generated sequence. Allocation was concealed until the intervention; however, participants and researchers were unblinded. The intervention group completed a 10-minute metaverse-based VR session, delivered by a consultant surgeon via 3D Organon’s Medverse platform on the PICO 4 Ultra headset. The control group completed a 10-minute prerecorded 2D animated lecture, accessed on their personal device. Participants then swapped to the other modality. Data were collected via Google Forms at 3 intervals (baseline, postintervention, and postcrossover) to assess confidence, spatial understanding, and knowledge (10-item multiple-choice questions). The analysis of nonparametric data utilized Wilcoxon signed-rank tests for within-group changes and Mann–Whitney *U* tests for between-group differences.

**Results:**

Twenty-four medical students from 11 United Kingdom and Irish medical schools participated. Adherence was 100%, with all participants completing their assigned 10-minute intervention and all assessment points. Ninety-two percent of participants (n=22) reported no prior tracheostomy anatomy teaching. Additionally, 83% (n=20) had no prior remote-synchronized VR anatomy teaching experience. Anatomical confidence improved significantly in the VR group compared with animation (mean change 1.58, SD 1.00 vs mean 0.50, SD 0.80, *P*=.01). Knowledge scores improved significantly in both groups (VR: mean 1.75, SD 1.54, *P*=.007; animation: mean 2.83, SD 1.70, *P*=.003), with no significant postintervention difference between groups (*P*=.46). VR participants reported significantly superior spatial understanding across all measured domains (all *P*≤.009). These included depth perception (3.75 vs 2.58, *P*=.009), appreciation of anatomy from different viewpoints (4.25 vs 2.33, *P*=.001), mental reconstruction from varying angles (3.83 vs 2.08, *P*=.002), and spatial depth supporting anatomical understanding (4.08 vs 2.08, *P*=.001). Following the completion of both modalities, participants rated VR as more engaging (mean 4.54, SD 0.78) and more educationally effective (mean 4.29, SD 0.95) than animation.

**Conclusions:**

Remote VR teaching is feasible and engaging and enhances spatial understanding compared to animation. While knowledge gains were comparable between modalities, VR improved learner confidence and perceived 3D comprehension. Hence, VR may represent a scalable adjunct or alternative to traditional anatomy teaching.

## Introduction

Anatomy teaching has traditionally relied on cadaveric dissection and physical classroom-based resources. However, inconsistent anatomy curricula across universities, reduced cadaver availability, and the shift toward blended and remote learning environments have highlighted limitations in the accessibility and flexibility of conventional methods [1]. 2D media, such as textbooks, surface anatomy diagrams, and videos, support factual knowledge acquisition but may offer less clarity when visualizing complex spatial relationships within the human body.

Virtual reality (VR) provides an immersive digital learning environment capable of rendering detailed 3D anatomical models that students can explore remotely and interactively. By allowing learners to visualize structures from multiple perspectives and manipulate their spatial orientation, VR offers potential advantages over passive modalities. Recent research suggests that VR can improve knowledge acquisition, retention, learning confidence, and conceptual comprehension, making it an appealing adjunct or alternative to traditional anatomy teaching, especially in settings where access to cadaveric facilities is limited [2,3].

Tracheostomy is among the most commonly performed procedures in individuals who are critically ill [4]. However, it is associated with a 40% to 50% rate of complications, which highlights that a strong anatomical understanding is essential for patient safety [5]. Knowledge of surface landmarks, anterior neck layers, and closely related vascular structures is crucial to avoiding complications during emergency and elective tracheostomy procedures. Despite this, many medical students do not encounter tracheostomies during clinical placements and have low confidence in tracheostomy care, highlighting a clear educational gap [6].

The growth of remote learning during and beyond the COVID-19 pandemic has created renewed interest in scalable digital approaches that support high-quality anatomical instruction outside the classroom. Platforms enabling synchronous VR teaching may therefore offer practical benefits in both accessibility and educational value. However, despite the increasing availability of VR technologies, evidence directly comparing VR to established 2D learning methods, such as animations, remains limited, especially for remote delivery. Whether VR provides measurable improvements in learning outcomes or confidence beyond those achievable with conventional digital resources is not yet fully understood.

To address this gap, we designed a randomized controlled trial comparing remote-synchronized VR teaching with a didactic animated anatomy lecture following a shared baseline lecture. Our primary objective was to evaluate whether VR enhances learners’ confidence and perceived spatial understanding of tracheostomy anatomy beyond what is achievable with a conventional 2D resource. In addition, we examined changes in anatomical knowledge to determine whether immersive VR offers measurable educational benefits when delivered remotely within an undergraduate medical curriculum.

## Methods

### Study Design

This was a prospective, randomized controlled pilot study comparing remote-synchronized VR teaching with a didactic animated anatomical lecture for learning tracheostomy anatomy. The study followed CONSORT-EHEALTH (Consolidated Standards of Reporting Trials of Electronic and Mobile Health Applications and Online Telehealth) reporting guidelines (Checklist 1). Due to the nature of the interventions, neither participants nor researchers were blinded to group allocation.

Following baseline assessment and a shared didactic lecture, participants were randomized in a 1:1 ratio using a computer-generated random sequence to receive either the VR session or the animation as their initial learning modality. Allocation was concealed until the commencement of the intervention phase. Outcome measures were collected immediately after the completion of this first assigned modality, permitting between-group comparisons of the primary intervention. Participants then crossed over to the alternate modality, ensuring that all participants experienced both teaching approaches during the study. Final assessments were completed after exposure to both modalities. The total study duration was approximately 60 minutes.

All participants attended a 20-minute didactic lecture delivered by a consultant surgeon, covering the four learning objectives: (1) identify tracheostomy surface landmarks, (2) describe the correct skin incision placement and orientation, (3) list the anterior neck layers to reach the trachea, and (4) recognize key structures to avoid and their relationships. These objectives were derived from the National Tracheostomy Safety Project e-learning module, which was developed by National Health Service England in collaboration with the Royal College of Anesthetists [7].

This shared lecture was intended to establish a common anatomical starting point before comparing the efficacy of the VR and 2D animation modalities. Consequently, the effect of the didactic lecture as a stand-alone educational intervention was not isolated or measured, as the preintervention and postintervention scores reflect the combined learning gain from the shared lecture followed by the assigned 10-minute intervention.

**Figure 1.**
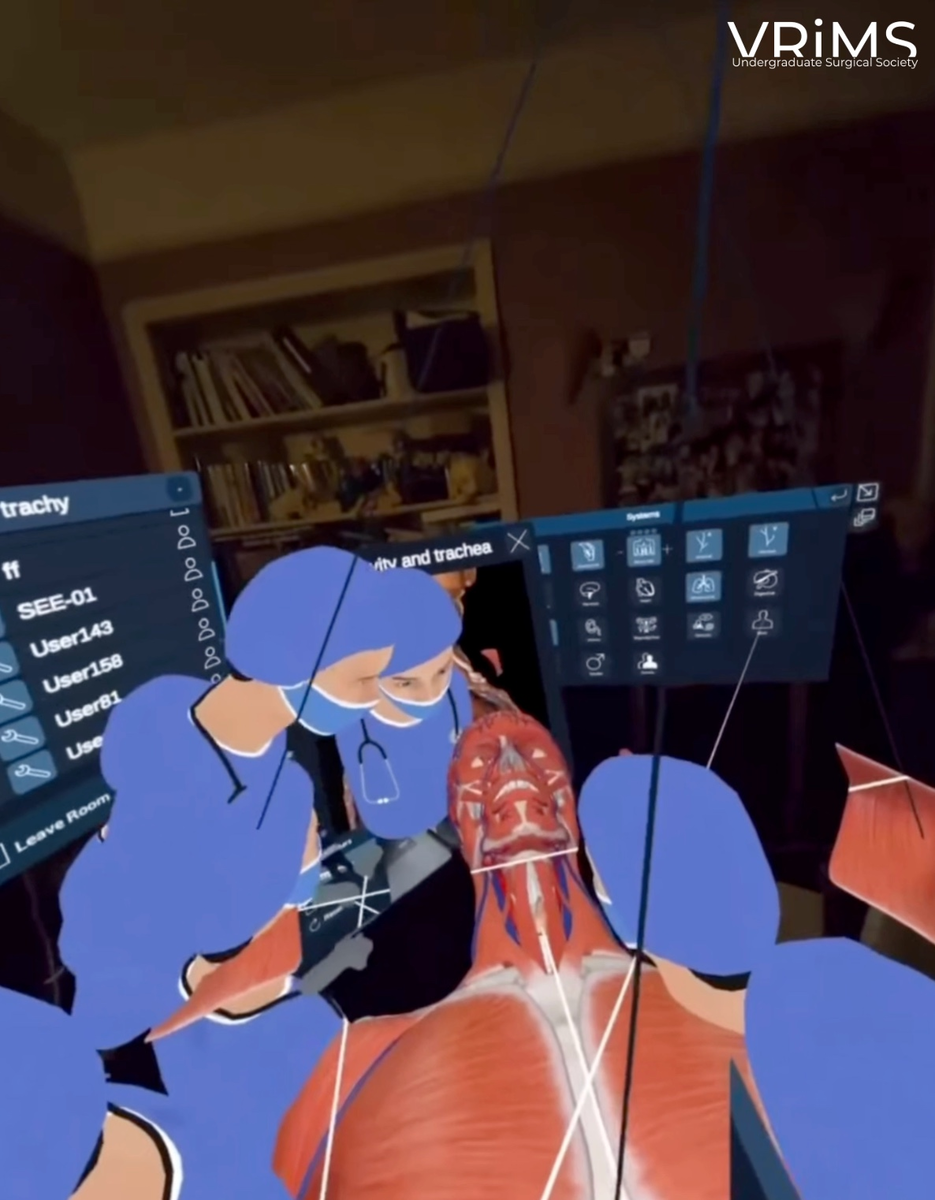
The Medverse platform interface using 3D Organon software, illustrating the synchronized virtual learning environment with active participant avatars.

Participants proceeded to their assigned learning modalities:

1. Intervention group: A 10-minute synchronized VR session in Medverse on 3D Organon, using a 6-degree-of-freedom headset (PICO 4 Ultra Enterprise Version). Medverse is a metaverse network module that allows participants to join multiuser online sessions. The consultant surgeon demonstrated the tracheostomy anatomy, including rotation and spatial navigation of cervical structures, with real-time verbal explanation (Figure 1).
2. Control group: Participants assigned to the control arm viewed a 10-minute publicly available YouTube video presenting key tracheostomy anatomy from a fixed 2D perspective with a verbal explanation [8]. The video was selected based on several criteria: it covered all 4 predefined learning objectives, was matched in duration to the VR session (10 min), and was created and narrated by an associate professor during head and neck surgery, ensuring accuracy and comparable instructional quality. At the time of the study, the video had accrued over 82,000 views and more than 1000 likes, reflecting its widespread use and positive reception within the medical education community. The use of a high-quality, widely accessed animation was intentional, providing a realistic and ecologically valid comparator, which reflects the type of resource that medical students would independently access for self-directed learning.

Participants completed 3 questionnaires on Google Forms (Google LLC): part 1 (Multimedia Appendix 1), part 2 (Multimedia Appendix 2), and part 3 (Multimedia Appendix 3), which were designed to assess confidence, spatial understanding, and anatomical knowledge. The questionnaires were developed specifically for this study and were not formally validated or pretested prior to deployment. The part 1 questionnaire was administered prior to the lecture, part 2 immediately after the participant’s assigned learning intervention, and part 3 at the end of the study once both learning modalities had been completed. The survey points in relation to the study structure are present in the Results section. Participant demographics collected were limited to current levels of medical training and institutional affiliation. Data regarding age, gender, and race/ethnicity were not recorded to prioritize participant anonymity and streamline the remote recruitment process via the VRiMS (Virtual Reality in Medicine and Surgery) network.

Confidence in performing a tracheostomy and in achieving the 4 predefined learning objectives was evaluated preintervention (part 1) and postintervention (part 2) using a self-reported 5-point Likert scale. Anatomical knowledge acquisition was measured using a 10-item best-of-5 multiple-choice assessment (Multimedia Appendix 4), with the same 10 questions administered in part 1 and part 2 to enable paired analysis of knowledge gain following the intervention. In addition, spatial understanding was assessed postintervention (part 2) using a self-reported 5-point Likert scale relating to participants’ ability to appreciate depth and orientation of anatomical structures, understand anatomy from different viewpoints, mentally reconstruct structures from varying perspectives, and recognize how spatial depth supports anatomical relationships. Part 3 also included items comparing the 2 modalities, asking participants to indicate which modality they found more engaging and which they perceived had greater educational impact on their learning.

The questionnaires were informed by the Cognitive Affective Model of Immersive Learning (CAMIL), which describes how immersive VR may enhance learning by increasing psychological presence and agency, thereby improving motivation, self-efficacy, and knowledge outcomes [9]. Preintervention items assessed prior anatomy teaching and previous exposure to VR to account for novelty and familiarity effects. Participants were also asked to rate their interest in learning anatomy using remote-synchronized VR, reflecting CAMIL’s emphasis on situational interest as a driver of engagement and learning. Confidence in tracheostomy anatomy was measured preintervention and postintervention as an indicator of self-efficacy. Telepresence was evaluated in part 3 once all participants had completed the VR modality. Items were adapted from the Temple Presence Inventory, a validated questionnaire designed to measure subjective experiences of presence, immersion, and realism in virtual environments [10]. This was used to assess the extent to which participants experienced a sense of “being there” within the virtual environment, perceived copresence with virtual characters, felt mentally immersed in the experience, and judged the environment and interactions as realistic. Each item taken from the Temple Presence Inventory was rated using a 5-point Likert scale instead of a 7-point scale for consistency. Perceived agency was assessed postintervention through an item evaluating the extent to which participants felt a strong sense of control and interaction with the virtual environment. These measures align with CAMIL’s proposed affective and cognitive pathways linking immersive experience to improved learning outcomes.

### Recruitment

The sampling frame consisted of medical students currently enrolled in the United Kingdom and Irish medical schools with access to the VRiMS Surgical Society communication channels. The study was conducted in a remote, distributed setting, with participants joining from their respective geographical locations via Zoom and the 3D Organon Medverse platform on November 2, 2025.

A convenience sampling method was employed. Recruitment was initiated 10 days prior to the study date through a multimodal approach: announcements via the VRiMS regional communication channels and direct mobilization through regional student leads at various medical faculties to ensure a broad geographic reach. Interested students completed a digital screening form to verify eligibility criteria (current medical student status and access to a stable internet connection).

As this was a pilot randomized controlled trial, a formal priori power calculation was not performed. A target sample size of 24 (12 per arm) was determined based on established recommendations for pilot studies [11], which suggest this size is sufficient to estimate the variance of primary outcomes and assess the feasibility of the remote synchronized VR protocol for future large-scale trials.

To minimize nonresponse bias, automated reminders were sent 24 hours prior. Of the 28 students who initially registered and were sent hardware, 4 were unable to attend due to technical or personal conflicts, resulting in a final analytical sample of 24. There was 0% attrition (dropout) once the session commenced, as all participants completed the final questionnaire.

### Analysis

Data were collected initially on Google Sheets (Google LLC) and transferred to Microsoft Excel (version 16.91, Microsoft Corp). These were then analyzed using SPSS version 31 (IBM).

Normality of continuous data was evaluated using the Shapiro-Wilk test. As the data were not normally distributed, nonparametric tests were applied. Paired preintervention and postintervention scores were compared using the Wilcoxon signed-rank test. Differences between groups were assessed using the Mann-Whitney *U* test. Categorical variables were compared using *χ*² tests or Fisher exact tests where appropriate. Likert scale data were summarized using means and SDs. Statistical significance was defined as *P*<.05.

### Ethical Considerations

This study was conducted remotely on November 2, 2025. Ethical approval for this study was granted by the Brighton and Sussex Medical School Research Governance and Ethics Committee (reference number ER/BSMS9GYI/1) in accordance with the Declaration of Helsinki and UK General Data Protection Regulation [12]. Participation was voluntary, with participants providing informed consent after reading the participant information sheet and agreeing for their data to be used toward this research study. All data were anonymized, and no identifiable information was collected. Participants did not receive financial compensation for their participation.

## Results

### Participant Characteristics and Baseline Data

**Figure 2.**
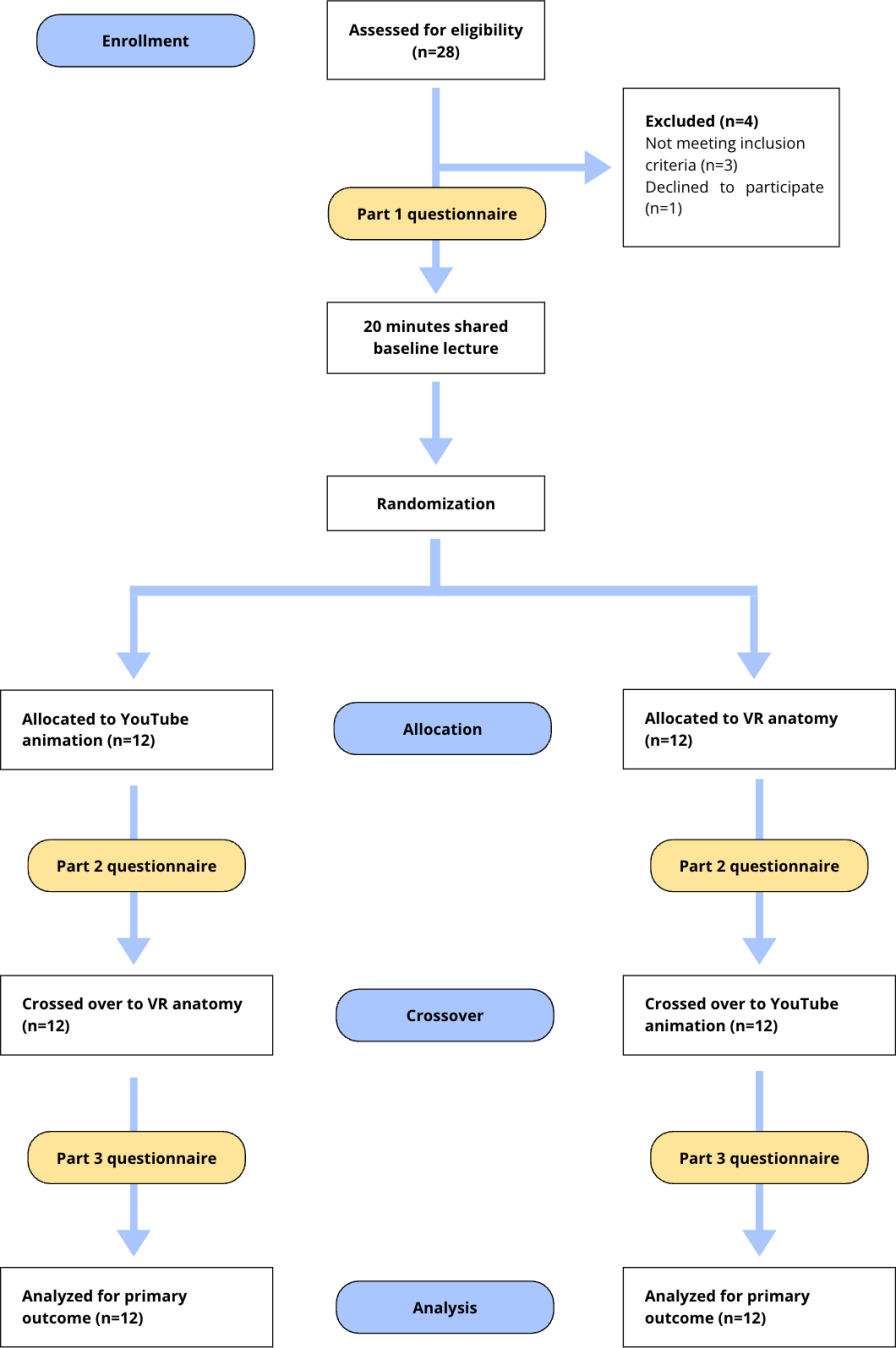
Adapted CONSORT (Consolidated Standards of Reporting Trials) 2025 flow diagram illustrating participant recruitment, randomization, and retention throughout the pilot study. VR: virtual reality.

**Figure 3.**
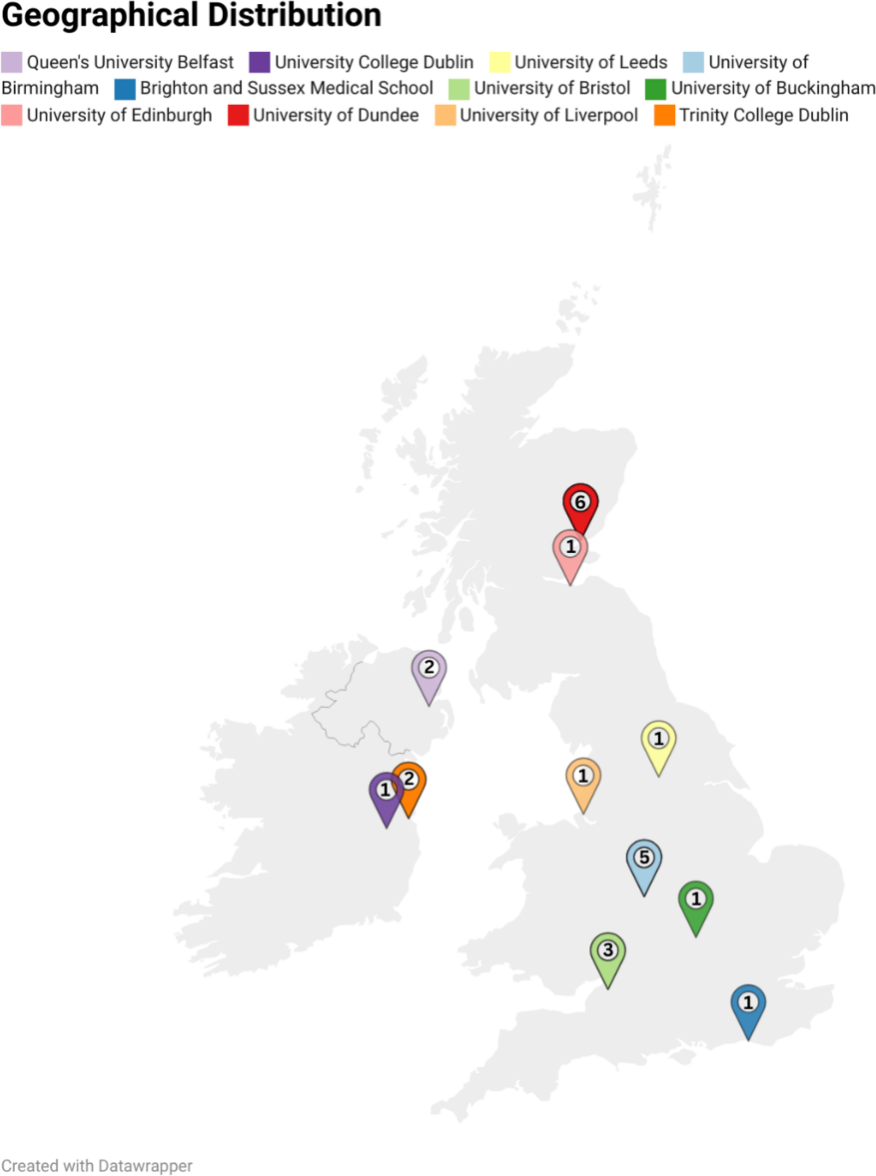
Geographical distribution of study participants across the United Kingdom and Ireland. Numerical values within the map markers indicate the frequency of participants per location.

A total of 28 medical students were initially recruited; however, 4 were excluded prior to participation (3 did not meet the inclusion criteria and 1 declined), resulting in 24 medical students (VR=12 and control=12) from 11 universities across 7 regions of the United Kingdom and the Republic of Ireland participating in the study. There was no loss to follow-up, and all 24 participants completed both learning modalities. Participant flow and the workshop schedule are illustrated in Figure 2. The majority of participants were in the preclinical or early clinical years: year 2 (n=6, 25%), year 3 (n=9, 38%), year 4 (n=7, 29%), year 5 (n=1, 4%), and year 6 (n=1, 4%). Geographically, participants were distributed across 7 regions, with the highest representation from Scotland (n=7) and the Midlands (n=5; Figure 3). Adherence was 100%, with all 24 participants completing both learning modalities and all assessment points, resulting in no missing data.

Baseline exposure to the topic was low; 92% (22/24) of participants reported 0 hours of prior anatomy teaching specific to tracheostomy. Similarly, 83% (20/24) had no prior experience with remote-synchronized VR anatomy teaching. Baseline interest in learning via remote-synchronized VR was high (mean 4.92, SD 0.28 on a 5-point Likert scale). Participants generally perceived their prior anatomy teaching on tracheostomy as insufficient (mean 1.92, SD 1.03). There were no significant differences between groups regarding baseline demographics and experiences, except for participant region (*P*=.20; Table 1).

**Table 1.** Baseline demographic and clinical characteristics of study participants by group, with intergroup statistical comparisons (*P* values).

Items and categories	VR^a^ (n=12), n	Non-VR (n=12), n	*P* value
Which year are you currently in?			.80
Year 2	4	2	
Year 3	4	5	
Year 4	3	4	
Year 5	0	1	
Year 6	1	0	
Which region are you in?			.10
Ireland	2	1	
South West	3	0	
Scotland	5	2	
North	0	2	
Midlands	1	4	
South East	0	2	
Northern Ireland	1	1	
Have you had any anatomy teaching on tracheostomy before?			>.99
No	11	10	
Yes (<1 h)	1	1	
Yes (1‐2 h)	0	1	
Have you participated in any remote-synchronized VR anatomy teaching before?			.22
No	12	9	
Yes (<1 h)	0	1	
Yes (1‐2 h)	0	1	
Yes (>2 h)	0	1	

a VR: virtual reality.

### Self-Reported Confidence and Learning Objectives

Self-reported confidence improved in both groups following the workshop; however, the VR group demonstrated consistently greater gains. Table 2 summarizes the changes in self-reported confidence across both groups. For confidence in understanding relevant anatomical structures, the VR group showed a significant increase from mean 2.00 (SD 0.953) pre-workshop to mean 3.58 (SD 0.900) post-workshop (+79%, *P*=.004), while the non-VR (animation) group exhibited a nonsignificant increase from mean 2.50 (SD 0.798) to mean 3.00 (SD 0.953) (+20%, *P*=.06). There were no statistically significant differences in baseline confidence (*P*=.22) between the groups prior to the workshop. Between-group analysis confirmed that VR training resulted in a significantly greater improvement in this domain (*P*=.01).

**Table 2.** Learning objectives and subgroup self-reported confidence scores (mean [SD]) before and after the intervention, including percentage change and 95% CIs.

Group	Pre-workshop, mean (SD)	Post-workshop, mean (SD)	% change	*P* value (within-group)	*P* value (between-group)
How confident are you in your understanding of anatomical structures relevant to tracheostomy?					.01
VR^a^	2.00 (0.953)	3.58 (0.900)	79	.004	
Non-VR	2.50 (0.798)	3.00 (0.953)	20	.06	
Learning objective 1—I can identify key surface landmarks for a tracheostomy					.01
VR	1.75 (0.754)	3.92 (0.900)	124	.002	
Non-VR	2.08 (0.793)	3.17 (0.937)	52	.01	
Learning objective 2—I understand where the skin incision is typically made for a tracheostomy					.10
VR	1.67 (0.985)	4.00 (0.953)	140	.002	
Non-VR	1.92 (0.793)	3.33 (1.073)	73	.008	
Learning objective 3—I can identify the key anterior neck layers leading to the trachea					.86
VR	2.17 (0.835)	3.50 (1.000)	61	.008	
Non-VR	1.92 (0.793)	3.50 (1.000)	82	.004	
Learning objective 4—I can identify the important structures to avoid during a tracheostomy					.17
VR	1.92 (0.996)	3.75 (0.866)	95	.003	
Non-VR	1.67 (0.651)	2.92 (1.165)	75	.006	

a VR: virtual reality.

Both groups demonstrated significant gains across all 4 learning objectives. For learning objective 1 (identifying key surface landmarks), the VR group improved from mean 1.75 (SD 0.754) to mean 3.92 (SD 0.900; +124%, *P*=.002), while the non-VR group increased from mean 2.08 (SD 0.793) to mean 3.17 (SD 0.937; +52%, *P*=.01). The between-group difference was statistically significant (*P*=.01), indicating a greater improvement with VR training. For learning objective 2 (understanding where the skin incision is typically made), VR participants showed a substantial increase from mean 1.67 (SD 0.985) to mean 4.00 (SD 0.953; +140%, *P*=.002), compared with an increase from mean 1.92 (SD 1.073) to mean 3.33 (SD 1.073; +73%, *P*=.008) in the non-VR group; however, this between-group difference was not statistically significant (*P*=.10). For learning objective 3 (identifying the anterior neck layers leading to the trachea), the VR group improved from mean 2.17 (SD 0.835) to mean 3.50 (SD 1.000; +61%, *P*=.008), and the non-VR group increased from mean 1.92 (SD 0.793) to mean 3.50 (SD 1.000; +82%, *P*=.004), with no significant difference between groups (*P*=.86). Finally, for learning objective 4 (identifying important structures to avoid), the VR group increased from mean 1.92 (SD 0.996) to mean 3.75 (SD 0.866; +95%, *P*=.003), while the non-VR group improved from mean 1.67 (SD 0.651) to mean 2.92 (SD 1.165; +75%, *P*=.006). Again, the between-group difference was not significant (*P*=.17).

### Spatial Understanding and Perspective

**Figure 4.**
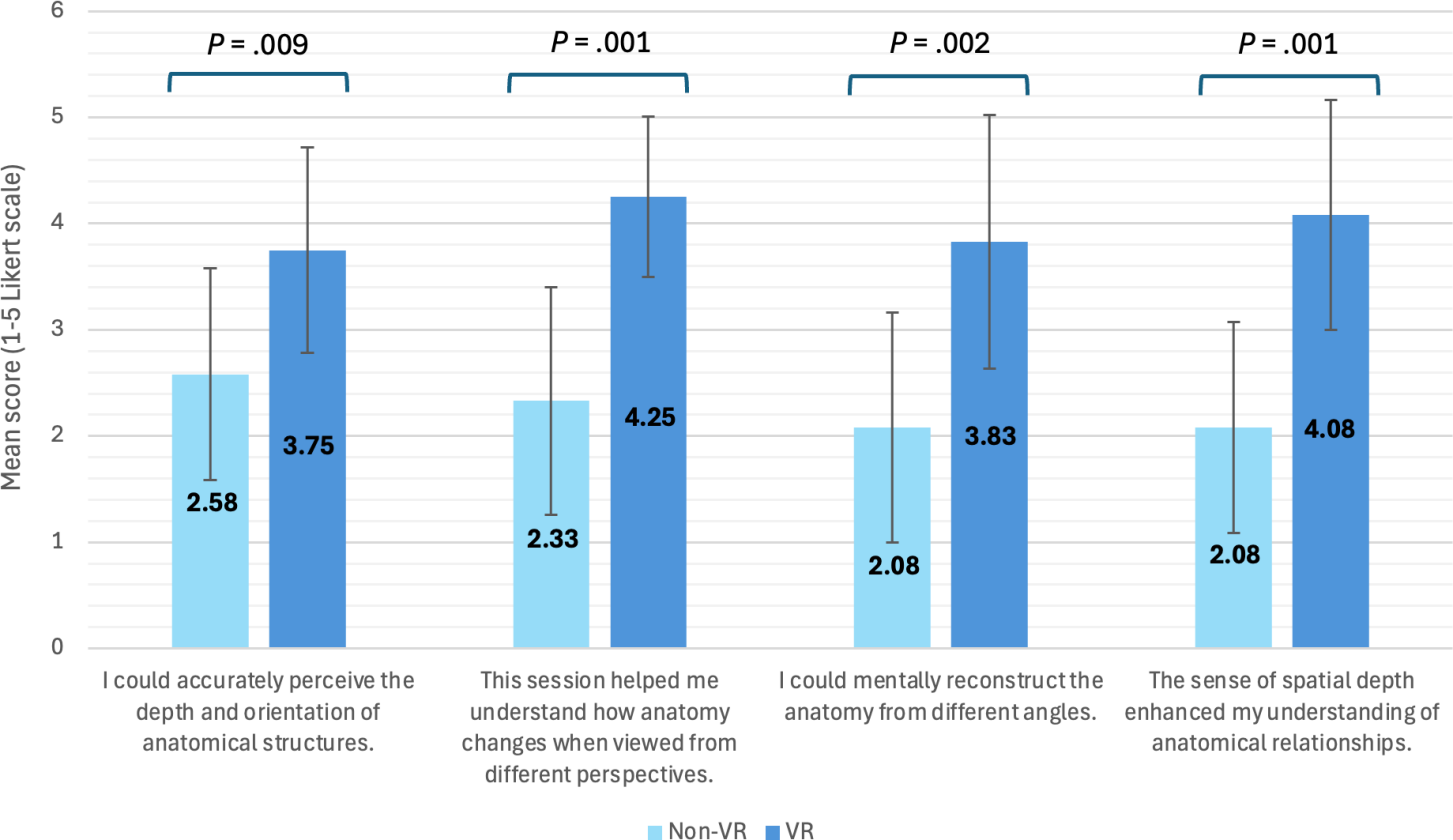
Comparative analysis of spatial understanding and perspective-taking capabilities between the virtual reality (VR) and non-VR study arms. Error bars represent SD.

Spatial understanding and perspective-taking scores were significantly higher in the VR group than in the non-VR group for all items assessed (Figure 4). Participants who received the VR modality reported a greater ability to accurately perceive the depth and orientation of anatomical structures (VR: mean 3.75, SD 0.965 vs non-VR: mean 2.58, SD 0.996; *P*=.009), a better understanding of how anatomy changes when viewed from different perspectives (VR: mean 4.25, SD 0.754 vs mean 2.33, SD 1.073; *P*=.001), and an improved ability to mentally reconstruct anatomy from different angles (VR: mean 3.83, SD 1.193 vs mean 2.08, SD 1.084; *P*=.002). VR participants also reported that the enhanced sense of spatial depth more strongly supported their anatomical understanding (VR: mean 4.08, SD 1.084 vs mean 2.08, SD 0.996; *P*=.001).

### 10-Item Anatomy Quiz

Anatomy knowledge was assessed using multiple-choice questions. There was no significant difference in baseline scores between the VR and animation groups (*P*=.50). Both groups demonstrated statistically significant improvements following the intervention. The animation group showed an increase from mean 3.92 (SD 2.392) to mean 6.75 (SD 1.913), representing a 72% gain (mean increase 2.83, SD 1.697; *P*=.003). The VR group improved from mean 4.33 (SD 2.188) to mean 6.08 (SD 2.503), corresponding to a 40% gain (mean increase 1.75, SD 1.545; *P*=.007). Although the animation group demonstrated a numerically greater improvement, the final posttest scores did not differ significantly between the groups (*P*=.46).

### Participant Feedback and Experience

After completing both modalities, participants provided feedback on their experience. Participant feedback on the learning experience was positive overall. Learners generally agreed that, compared to the animation, VR supported more effective learning (mean 4.29, SD 0.95) and was more engaging (mean 4.54, SD 0.78). Participants mostly agreed that they felt a strong sense of control within the virtual environment (mean 4.04, SD 0.94). They also reported a moderate-to-strong sense of shared presence (mean 3.79, SD 1.00) and a high level of mental immersion (mean 4.12, SD 0.83).

## Discussion

### Principal Results

Our randomized controlled trial demonstrates that a remote, immersive VR anatomy session has the potential to significantly enhance medical students’ confidence and spatial understanding of tracheostomy anatomy compared to a conventional 2D animation. Participants in the VR group reported larger gains in self-efficacy for identifying cervical landmarks and structures, partially consistent with our primary hypothesis. Significant between-group differences in confidence were observed for overall anatomical understanding and surface landmark identification, though not for all individual learning objectives. Crucially, VR also conferred a marked advantage in perceived 3D comprehension; students felt far better able to appreciate depths, orientations, and anatomical relationships after the VR experience than after viewing a nonimmersive animation. These findings indicate that the interactive 3D visualization and immersion afforded by VR can address known challenges of 2D anatomy resources (eg, difficulty visualizing complex spatial relationships) [2]. Notably, both VR and animation groups showed significant improvements on the factual anatomy quiz, with no difference in postintervention scores between modalities. This suggests that for short-term knowledge acquisition, VR was at least as effective as a didactic animated anatomical lecture, while providing added benefits in learner confidence and conceptual understanding.

### Comparison With Prior Work

Our findings are consistent with a growing body of literature evaluating the role of immersive VR in medical education. Prior studies have repeatedly demonstrated that while VR does not always outperform traditional methods in terms of short-term factual knowledge acquisition, it offers clear advantages in learner engagement, motivation, and perceived educational value. For example, one study reported no significant difference in neuroanatomy test scores between a VR module and textbook learning; yet the VR group found the experience significantly more enjoyable and motivating [13]. Similarly, another study found no differences in assessment outcomes among students learning skull anatomy via VR, augmented reality, or tablet-based applications, but VR was consistently rated as more immersive and engaging. These findings suggest that VR’s educational value may lie less in improving immediate test performance and more in enhancing affective and cognitive factors known to support deeper learning. This interpretation is reinforced by a recent meta-analysis of 15 randomized controlled trials, which demonstrated a substantial and consistent increase in student interest and satisfaction with VR-based learning (pooled standardized mean difference≈0.77 in favor of VR) [14]. Our results align closely with these observations, as participants overwhelmingly reported the VR session to be more engaging and perceived it as a more effective learning experience than animation, despite similar objective knowledge gains.

### Spatial Understanding

Beyond engagement, VR’s strengths appear particularly pronounced in supporting spatial understanding, a core competency in anatomy and surgery. Improved spatial orientation with VR has been objectively demonstrated across multiple surgical and anatomical contexts. For instance, neurosurgical trainees using 3D VR models identified aneurysm anatomy faster and preferred VR over 2D images [15]. A substantial body of research has established spatial ability as a key determinant of success in anatomy learning and surgical performance, underscoring the educational relevance of technologies that enhance spatial cognition [16,17]. The stereoscopic depth cues and interactivity inherent to VR are central to these advantages. Wainman et al [18] showed that a true 3D VR view enabled significantly better anatomical structure identification than identical 2D images, whereas blocking depth perception erased the VR benefit. This finding highlights that VR’s advantage is not simply due to novelty or visual appeal but rather due to its capacity to convey depth, orientation, and spatial relationships in a way that flat representations cannot. Our study extends this evidence to a fully remote, synchronized teaching context. Despite participants being geographically dispersed, VR facilitated an interactive, spatially rich learning experience that more closely approximated hands-on anatomical exploration than passive video-based instructions. This suggests that VR can preserve key educational benefits of in-person anatomy teaching even when delivered remotely, with particular value for topics, such as tracheostomy anatomy, where accurate spatial understanding is essential for clinical safety.

### Cognitive Affective Model of Immersive Learning

Our findings suggest that the VR experience influenced multiple key factors in line with CAMIL. Beyond boosting students’ confidence (self-efficacy), the heightened sense of presence (feeling of being inside the virtual world) and agency (control over the virtual environment), both of which were directly measured in this study, likely made the lesson more engaging and novel for learners. This immersive engagement would naturally spark greater situational interest in the subject matter and increase intrinsic motivation to learn. CAMIL posits that such presence-driven interest and motivation boosts are fundamental advantages of VR, as the intense engagement can make learning feel more effortless and enjoyable for students. These observations align with our results, where the VR group reported being more interested and invested in the learning activity than traditional methods. Another critical factor influenced by the VR intervention was self-efficacy—students’ belief in their ability to perform the learning tasks. The increase in self-reported confidence we observed is consistent with CAMIL’s claim that immersive environments can strengthen learners’ self-efficacy. By giving students a high degree of control and a first-person role in the simulation, the VR provided experiences that reinforced their sense of competence [19]. Furthermore, while not directly measured in this study, CAMIL would predict that the immersive scenario likely promoted a feeling of embodiment, meaning learners felt physically and mentally present in the virtual situation. CAMIL identifies embodiment as another affective factor that can enhance learning by engaging sensory and motor processes: when students can interact with 3D objects or move within a scene, it activates more neural pathways and can deepen understanding [20]. In our study’s VR module, this embodiment might have helped students internalize concepts and skills more effectively—for instance, by virtually “learning by doing,” which can strengthen memory traces and procedural knowledge. Together, the boosts in self-efficacy and the sense of embodiment likely contributed to the improved learning confidence and competence we documented in the VR group.

### Limitations

This pilot study has several limitations that temper the interpretation of the results. First, the sample size was modest (24 students), which limits statistical power and the generalizability of the findings. The participants were also not a random cross-section of all medical students—they were recruited through a VR-focused society and had a very high baseline interest in VR learning (mean ~4.9 on a 5-point scale). This self-selection could introduce bias, as enthusiastic students might rate the VR experience more favorably due to novelty or positive expectations. Participant heterogeneity also represents a limitation. Surgical training is inherently individualized, and variability in prior knowledge, experience, and stage of training may have influenced both baseline performance and the magnitude of improvement observed. While this variability represents a limitation in the standardization of outcomes, it reflects real-world training environments in which cohorts are diverse and exposure is varied.

A key limitation of this study is the confounding variable between arms: the VR group received live, synchronous instruction from a consultant, while the control group viewed a passive, prerecorded animation. While this design reflects the authentic, real-world deployment of VR as a “complete educational package,” it prevents the effects of the immersive modality from being fully disentangled from the influence of real-time instructor presence. Consequently, the superior outcomes in the VR group may be attributed to either the technology itself or the heightened interactivity of live teaching.

Another limitation of this pilot study is the absence of detailed baseline demographic data, including age, gender, and ethnicity. While our sample consisted of medical students from across the United Kingdom and Ireland, the lack of these characteristics prevents an assessment of whether the educational impact of VR varies across different demographic groups. Future large-scale trials should incorporate comprehensive demographic screening to ensure the generalizability of these findings to a diverse student population.

Our knowledge assessment was limited to a short 10-item multiple-choice quiz administered immediately after the intervention. In particular, with a best-of-5 format, some students’ score improvements could reflect random guessing or test familiarity rather than true learning gains. The lack of a delayed post test means we could not evaluate knowledge retention beyond the immediate session. Third, the measures of confidence and spatial understanding were based on self-reported Likert scale ratings. Such subjective outcomes might be influenced by the excitement of using a novel VR tool, and there was no objective test of spatial skills to corroborate the perceived improvements. In addition, the use of a 5-point Likert scale introduces a ceiling effect, with many postintervention confidence scores clustering at the upper end of the scale. This perhaps reduced the sensitivity of between-group comparisons, particularly for confidence outcomes, and may have contributed to the absence of statistically significant differences despite larger absolute gains in the VR group.

There are practical constraints to consider: VR hardware and setup requirements. In our study, all participants were provided with VR headsets, but in real-world settings, the cost and logistics of ensuring every student has access to a compatible device could be a barrier. These factors highlight that while VR is a promising tool, careful attention must be paid to equipment access, training, and user comfort when deploying it at scale.

### Future Directions

An important advantage highlighted by this study is the scalability and reach of VR-based anatomy teaching. This has important implications for remote learning and for medical schools operating in resource-limited settings. VR technology offers a means of standardizing access to high-quality anatomy education by virtually delivering detailed, cadaveric-equivalent 3D anatomical models to students who lack consistent access to dissection facilities. In environments with limited anatomy laboratories, restricted cadaver availability, or a shortage of specialist instructors, immersive digital platforms represent a practical and potentially cost-effective alternative that does not rely on physical specimens or extensive infrastructure [21]. VR has been proposed as a strategy to reduce educational inequities, enabling institutions in lower-resource settings to bypass traditional infrastructure constraints through the adoption of virtual anatomy laboratories. Our findings support this perspective, demonstrating that immersive anatomy teaching can be delivered effectively at scale and across diverse locations. Importantly, these results reinforce the view that the greatest educational benefit is likely achieved not by replacing conventional approaches but by integrating VR with traditional methods, allowing each modality to complement the other and collectively enhance anatomy education [22].

### Conclusion

This study demonstrates that remote-synchronous VR is a feasible and highly effective modality for teaching the complex 3D anatomy of tracheostomy. While it appears noninferior to didactic animated anatomical lectures for factual recall, it is significantly superior for developing spatial understanding and learner confidence. As medical curricula continue to evolve toward digital and blended learning models, VR offers a powerful adjunct to traditional teaching, particularly for high-risk procedures where an understanding of spatial depth is critical for patient safety. Future research should focus on larger cohorts and longitudinal follow-ups to assess the retention of these spatial skills over time.

## Supplementary material

10.2196/93092 Multimedia Appendix 1 Part 1 questionnaire.

10.2196/93092 Multimedia Appendix 2 Part 2 questionnaire.

10.2196/93092 Multimedia Appendix 3 Part 3 questionnaire.

10.2196/93092 Multimedia Appendix 4 Anatomy multiple-choice questions.

10.2196/93092 Checklist 1 CONSORT checklist.

## Abbreviations

CAMIL: Cognitive Affective Model of Immersive Learning
CONSORT: Consolidated Standards of Reporting Trials
VR: virtual reality
VRiMS: Virtual Reality in Medicine and Surgery
