# Supplementary material for: Metaverse-Based Virtual Reality for Remote Anatomy Education: Pilot Randomized Controlled Trial
Source: JMIR Form Res. 2026 May 19;10:e93092. doi: 10.2196/93092 (PMC13186309; doi:10.2196/93092)
Supplement: Multimedia Appendix 1 [file formative-v10-e93092-s001.docx]

## **Appendix A - Part 1 Questionnaire**

1. **Which medical school do you go to?**
2. **Which year are you currently in?**

- Year 1
- Year 2
- Year 3
- Year 4
- Year 5
- Year 6
- Others (e.g. intercalating)

1. **Have you had any anatomy teaching on tracheostomy before?**

- No
- Yes (< 1 hr)
- Yes (1 - 2 hrs)
- Yes (> 2 hrs)

1. **Have you participated in any remote synchronized VR anatomy teaching before?**

- No
- Yes (< 1 hr)
- Yes (1 - 2 hrs)
- Yes (> 2 hrs)

1. **I am interested in learning anatomy using remote synchronized VR.**

- Scale (1 = strongly disagree, 5 = strongly agree)

1. **I believe that I have received sufficient anatomy teaching on tracheostomy in medical school.**

- Scale (1 = strongly disagree, 5 = strongly agree)

1. **How confident are you in your understanding of anatomical structures relevant to tracheostomy?**

- Scale (1 = strongly disagree, 5 = strongly agree)

1. **I can identify key surface landmarks for a tracheostomy.**

- Scale (1 = strongly disagree, 5 = strongly agree)

1. **I understand where the skin incision is typically made for a tracheostomy.**

- Scale (1 = strongly disagree, 5 = strongly agree)

1. **I can identify the key anterior neck layers leading to the trachea.**

- Scale (1 = strongly disagree, 5 = strongly agree)

1. **I can identify the important structures to avoid during a tracheostomy.**

- Scale (1 = strongly disagree, 5 = strongly agree)
