# Supplementary material for: Metaverse-Based Virtual Reality for Remote Anatomy Education: Pilot Randomized Controlled Trial
Source: JMIR Form Res. 2026 May 19;10:e93092. doi: 10.2196/93092 (PMC13186309; doi:10.2196/93092)
Supplement: Multimedia Appendix 2 [file formative-v10-e93092-s002.docx]

## Appendix B - Part 2 Questionnaire

1. **How confident are you in your understanding of anatomical structures relevant to tracheostomy?**

- ​​Scale (1 = strongly disagree, 5 = strongly agree)

1. **I can identify key surface landmarks for a tracheostomy.**

- Scale (1 = strongly disagree, 5 = strongly agree)

1. **I understand where the skin incision is typically made for a tracheostomy.**

- Scale (1 = strongly disagree, 5 = strongly agree)

1. **I can identify the key anterior neck layers leading to the trachea.**

- Scale (1 = strongly disagree, 5 = strongly agree)

1. **I can identify the important structures to avoid during a tracheostomy.**

- Scale (1 = strongly disagree, 5 = strongly agree)

1. **I could accurately perceive the depth and orientation of anatomical structures.**

- Scale (1 = strongly disagree, 5 = strongly agree)

1. **This session helped me understand how anatomy changes when viewed from different perspectives.**

- Scale (1 = strongly disagree, 5 = strongly agree)

1. **I could mentally reconstruct the anatomy from different angles.**

- Scale (1 = strongly disagree, 5 = strongly agree)

1. **The sense of spatial depth enhanced my understanding of anatomical relationships.**

- Scale (1 = strongly disagree, 5 = strongly agree)
