# Supplementary material for: Metaverse-Based Virtual Reality for Remote Anatomy Education: Pilot Randomized Controlled Trial
Source: JMIR Form Res. 2026 May 19;10:e93092. doi: 10.2196/93092 (PMC13186309; doi:10.2196/93092)
Supplement: Multimedia Appendix 3 [file formative-v10-e93092-s003.docx]

**Appendix C - Part 3 Questionnaire**

1. **I learned more effectively with the VR experience compared to the animation.**

- Scale (1 = strongly disagree, 5 = strongly agree)

1. **The VR session was more engaging than the animation.**

- Scale (1 = strongly disagree, 5 = strongly agree)

1. **During the VR session, I felt a strong sense of control and interaction with the virtual environment.**

- Scale (1 = strongly disagree, 5 = strongly agree)

1. **During the VR session, how much did it seem as if you and the people you saw were together in the same place?**

- Scale (1 = not at all, 5 = very much)

1. **During the VR session, to what extent did you feel mentally immersed in the experience?**

- Scale (1 = not at all, 5 = very much)

1. **How could this VR anatomy teaching be improved for future students?**

- Short answer
