# Supplementary material for: Metaverse-Based Virtual Reality for Remote Anatomy Education: Pilot Randomized Controlled Trial
Source: JMIR Form Res. 2026 May 19;10:e93092. doi: 10.2196/93092 (PMC13186309; doi:10.2196/93092)
Supplement: Multimedia Appendix 4 [file formative-v10-e93092-s004.docx]

**Appendix D - Anatomy Multiple Choice Questions**

**1. What is the ideal site for tracheostomy?**

A. 1st and 2nd rings

B. 2nd and 3rd rings

C. 3rd and 4th rings

D. 4th and 5th rings

E. Below 5th ring

Answer = B

**2. Which of the following correctly lists the layers from superficial to deep during tracheostomy?**

A. Skin → Platysma → Deep fascia → Trachea

B. Skin → Deep fascia → Strap muscles → Trachea

C. Skin → Platysma → Investing layer of deep fascia → Strap muscles → Pretracheal fascia → Trachea

D. Skin → Strap muscles → Pretracheal fascia → Trachea

E. Skin → Platysma → Carotid sheath → Trachea

Answer = C

**3. The carotid sheath, which contains critical neurovascular structures, is located in which anatomical position relative to the trachea?**

A. Directly anterior to the trachea.

B. Directly posterior to the trachea.

C. Inferior and medial to the trachea.

D. Superior and lateral to the trachea.

E. Posterolateral to the trachea.

Answer = E

**4. To locate the ideal site for a standard tracheostomy incision, which of the following is the most reliable pair of anatomical landmarks?**

A. The hyoid bone and the angle of the mandible.

B. The thyroid cartilage and the suprasternal notch.

C. The cricoid cartilage and the clavicle.

D. The sternal notch and the manubrium.

E. The cricoid cartilage and the suprasternal notch.

Answer = E

**5. A surgeon is attempting to identify the second and third tracheal rings for an incision. Which surface landmark is most helpful in this identification?**

A. The thyroid notch.

B. The hyoid bone.

C. The thyroid gland's lateral lobes.

D. The cricoid cartilage.

E. The sternocleidomastoid muscle's medial border.

Answer = D

**6. The recurrent laryngeal nerves are a key structure to avoid. Where are these nerves typically located in relation to the trachea and esophagus?**

A. Anteriorly, running directly over the tracheal rings.

B. Posteriorly, in the groove between the trachea and the esophagus.

C. Laterally, enclosed within the carotid sheath.

D. Superiorly, near the cricoid cartilage.

E. They are not a concern in a standard low-cervical tracheostomy.

Answer = B

**7. Which midline structure commonly overlies the 2nd to 4th tracheal rings and may need to be divided or retracted during tracheostomy?**

A. Brachiocephalic vein

B. Common carotid artery

C. Thyroid isthmus

D. Inferior thyroid artery

E. Recurrent laryngeal nerve

Answer = C

**8. Which pair of strap muscles are retracted laterally during tracheostomy to expose the trachea?**

A. Sternohyoid and sternothyroid.

B. Sternothyroid and omohyoid.

C. Thyrohyoid and omohyoid.

D. Sternohyoid and thyrohyoid.

E. Cricothyroid and omohyoid.
Answer = A

**9. Which fascial layer encloses the infrahyoid (strap) muscles and lies superficial to the trachea?**

A. Prevertebral fascia

B. Investing layer of deep cervical fascia

C. Carotid sheath

D. Pretracheal fascia

E. Buccopharyngeal fascia

Answer = D

**10. Which structure marks the superior limit of the trachea and can be palpated just below the thyroid cartilage?**

A. Suprasternal notch

B. Cricoid cartilage

C. Hyoid bone

D. Jugular notch

E. Thyroid isthmus

Answer = B
