# Supplementary material for: Metaverse-Based Virtual Reality for Remote Anatomy Education: Pilot Randomized Controlled Trial
Source: JMIR Form Res. 2026 May 19;10:e93092. doi: 10.2196/93092 (PMC13186309; doi:10.2196/93092)
Supplement: Checklist 1 [file formative-v10-e93092-s005.pdf]

CONSORT-EHEALTH (V 1.6.1) - Submission/Publication Form

The CONSORT-EHEALTH checklist is intended for authors of randomized trials evaluating web-based and Internet-based applications/interventions, including mobile interventions, electronic games (incl multiplayer games), social media, certain telehealth applications, and other interactive and/or networked electronic applications. Some of the items (e.g. all subitems under item 5 - description of the intervention) may also be applicable for other study designs.

The goal of the CONSORT EHEALTH checklist and guideline is to be
a) a guide for reporting for authors of RCTs,
b) to form a basis for appraisal of an ehealth trial (in terms of validity)

CONSORT-EHEALTH items/subitems are MANDATORY reporting items for studies published in the Journal of Medical Internet Research and other journals / scientific societies endorsing the checklist.

Items numbered 1., 2., 3., 4a., 4b etc are original CONSORT or CONSORT-NPT (non-pharmacologic treatment) items.
Items with Roman numerals (i., ii, iii, iv etc.) are CONSORT-EHEALTH extensions/clarifications.

As the CONSORT-EHEALTH checklist is still considered in a formative stage, we would ask that you also RATE ON A SCALE OF 1-5 how important/useful you feel each item is FOR THE PURPOSE OF THE CHECKLIST and reporting guideline (optional).

Mandatory reporting items are marked with a red *.
In the textboxes, either copy & paste the relevant sections from your manuscript into this form - please include any quotes from your manuscript in QUOTATION MARKS,
or answer directly by providing additional information not in the manuscript, or elaborating on why the item was not relevant for this study.

YOUR ANSWERS WILL BE PUBLISHED AS A SUPPLEMENTARY FILE TO YOUR PUBLICATION IN JMIR AND ARE CONSIDERED PART OF YOUR PUBLICATION (IF ACCEPTED).
Please fill in these questions diligently. Information will not be copyedited, so please use proper spelling and grammar, use correct capitalization, and avoid abbreviations.

DO NOT FORGET TO SAVE AS PDF _AND_ CLICK THE SUBMIT BUTTON SO YOUR ANSWERS ARE IN OUR DATABASE !!!

Citation Suggestion (if you append the pdf as Appendix we suggest to cite this paper in the caption):
Eysenbach G, CONSORT-EHEALTH Group
[illegible]

You're editing your response. Sharing this URL allows others to also edit your response.

FILL IN A NEW RESPONSE

URL: http://www.jmir.org/2011/4/e126/
doi: 10.2196/jmir.1923
PMID: 22209829

jasonha2429@gmail.com Switch accounts

Not shared

Resubmit to save

* Indicates required question

Your name *

First Last

Jason Ha

Primary Affiliation (short), City, Country *

University of Toronto, Toronto, Canada

University of Bristol, Bristol, United Kingdom

Your e-mail address *

abc@gmail.com

jasonylha@gmail.com

Title of your manuscript *

Provide the (draft) title of your manuscript.

Metaverse-based Virtual Reality for Remote Anatomy Education: A Pilot Randomized Controlled Trial

You're editing your response. Sharing this URL allows others to also edit your response.

FILL IN A NEW RESPONSE

Name of your App/Software/Intervention *

If there is a short and a long/alternate name, write the short name first and add the long name in brackets.

3D Organon Medverse

Evaluated Version (if any)

e.g. "V1", "Release 2017-03-01", "Version 2.0.27913"

2025.2.6

Language(s) *

What language is the intervention/app in? If multiple languages are available, separate by comma (e.g. "English, French")

English

URL of your Intervention Website or App

e.g. a direct link to the mobile app on app in appstore (itunes, Google Play), or URL of the website. If the intervention is a DVD or hardware, you can also link to an Amazon page.

https://www.3dorganon.com/

URL of an image/screenshot (optional)

Your answer

You're editing your response. Sharing this URL allows others to also edit your response.

FILL IN A NEW RESPONSE

Accessibility *

Can an enduser access the intervention presently?

- ○ access is free and open
- ○ access only for special usergroups, not open
- ◉ access is open to everyone, but requires payment/subscription/in-app purchases
- ○ app/intervention no longer accessible
- ○ Other:

Primary Medical Indication/Disease/Condition *

e.g. "Stress", "Diabetes", or define the target group in brackets after the condition, e.g. "Autism (Parents of children with)", "Alzheimers (Informal Caregivers of)"

Medical Students

Primary Outcomes measured in trial *

comma-separated list of primary outcomes reported in the trial

Anatomical knowledge and confidence.

Secondary/other outcomes

Are there any other outcomes the intervention is expected to affect?

Spatial understanding, level of immersion, level of presence, level of realism, perceived agency.

You're editing your response. Sharing this URL allows others to also edit your response.

FILL IN A NEW RESPONSE

Recommended "Dose" *

What do the instructions for users say on how often the app should be used?

- ○ Approximately Daily
- ○ Approximately Weekly
- ○ Approximately Monthly
- ○ Approximately Yearly
- ○ "as needed"
- ◉ Other: Once during the pilot trial.

Approx. Percentage of Users (starters) still using the app as recommended after 3 months *

- ◉ unknown / not evaluated
- ○ 0-10%
- ○ 11-20%
- ○ 21-30%
- ○ 31-40%
- ○ 41-50%
- ○ 51-60%
- ○ 61-70%
- ○ 71%-80%
- ○ 81-90%
- ○ 91-100%
- ○ Other:

You're editing your response. Sharing this URL allows others to also edit your response.

FILL IN A NEW RESPONSE

Overall, was the app/intervention effective? *

- ( ) yes: all primary outcomes were significantly better in intervention group vs control
- (•) partly: SOME primary outcomes were significantly better in intervention group vs control
- ( ) no statistically significant difference between control and intervention
- ( ) potentially harmful: control was significantly better than intervention in one or more outcomes
- ( ) inconclusive: more research is needed
- ( ) Other:

Article Preparation Status/Stage *

At which stage in your article preparation are you currently (at the time you fill in this form)

- ( ) not submitted yet - in early draft status
- ( ) not submitted yet - in late draft status, just before submission
- ( ) submitted to a journal but not reviewed yet
- (•) submitted to a journal and after receiving initial reviewer comments
- ( ) submitted to a journal and accepted, but not published yet
- ( ) published
- ( ) Other:

You're editing your response. Sharing this URL allows others to also edit your response.

FILL IN A NEW RESPONSE

Journal *

If you already know where you will submit this paper (or if it is already submitted), please provide the journal name (if it is not JMIR, provide the journal name under "other")

- ○ not submitted yet / unclear where I will submit this
- ○ Journal of Medical Internet Research (JMIR)
- ○ JMIR mHealth and UHealth
- ○ JMIR Serious Games
- ○ JMIR Mental Health
- ○ JMIR Public Health
- ◉ JMIR Formative Research
- ○ Other JMIR sister journal
- ○ Other:

Is this a full powered effectiveness trial or a pilot/feasibility trial? *

- ◉ Pilot/feasibility
- ○ Fully powered

Manuscript tracking number *

If this is a JMIR submission, please provide the manuscript tracking number under "other" (The ms tracking number can be found in the submission acknowledgement email, or when you login as author in JMIR. If the paper is already published in JMIR, then the ms tracking number is the four-digit number at the end of the DOI, to be found at the bottom of each published article in JMIR)

- ○ no ms number (yet) / not (yet) submitted to / published in JMIR
- ◉ Other: 93092

You're editing your response. Sharing this URL allows others to also edit your response.

FILL IN A NEW RESPONSE

TITLE AND ABSTRACT

1a) TITLE: Identification as a randomized trial in the title

1a) Does your paper address CONSORT item 1a? *

I.e does the title contain the phrase "Randomized Controlled Trial"? (if not, explain the reason under "other")

- (●) yes
- ( ) Other:

1a-i) Identify the mode of delivery in the title

Identify the mode of delivery. Preferably use “web-based” and/or “mobile” and/or “electronic game” in the title. Avoid ambiguous terms like “online”, “virtual”, “interactive”. Use “Internet-based” only if Intervention includes non-web-based Internet components (e.g. email), use “computer-based” or “electronic” only if offline products are used. Use “virtual” only in the context of “virtual reality” (3-D worlds). Use “online” only in the context of “online support groups”. Complement or substitute product names with broader terms for the class of products (such as “mobile” or “smart phone” instead of “iphone”), especially if the application runs on different platforms.

	1	2	3	4	5	
subitem not at all important	( )	( )	( )	( )	(●)	essential

Clear selection

Does your paper address subitem 1a-i? *

Copy and paste relevant sections from manuscript title (include quotes in quotation marks "like this" to indicate direct quotes from your manuscript), or elaborate on this item by providing additional information not in the ms, or briefly explain why the item is not applicable/relevant for your study

[illegible]

You're editing your response. Sharing this URL allows others to also edit your response. FILL IN A NEW RESPONSE

1a-ii) Non-web-based components or important co-interventions in title

Mention non-web-based components or important co-interventions in title, if any (e.g., “with telephone support”).

	1	2	3	4	5	
subitem not at all important	(selected)	○	○	○	○	essential

Clear selection

Does your paper address subitem 1a-ii?

Copy and paste relevant sections from manuscript title (include quotes in quotation marks "like this" to indicate direct quotes from your manuscript), or elaborate on this item by providing additional information not in the ms, or briefly explain why the item is not applicable/relevant for your study

Our intervention is web/platform-based (3D Organon Medverse accessed via VR headset over internet). There are no additional non-web-based components or important co-interventions beyond the shared baseline lecture, which is part of both arms equally.

1a-iii) Primary condition or target group in the title

Mention primary condition or target group in the title, if any (e.g., “for children with Type I Diabetes”) Example: A Web-based and Mobile Intervention with Telephone Support for Children with Type I Diabetes: Randomized Controlled Trial

	1	2	3	4	5	
subitem not at all important	(selected)	○	○	○	○	essential

Clear selection

You're editing your response. Sharing this URL allows others to also edit your response.

FILL IN A NEW RESPONSE

Does your paper address subitem 1a-iii? *

Copy and paste relevant sections from manuscript title (include quotes in quotation marks "like this" to indicate direct quotes from your manuscript), or elaborate on this item by providing additional information not in the ms, or briefly explain why the item is not applicable/relevant for your study

The study targets anatomy education broadly for healthcare learners. Being a medical student was not an explicit inclusion criterion; findings are applicable to the wider healthcare profession.

1b) ABSTRACT: Structured summary of trial design, methods, results, and conclusions

NPT extension: Description of experimental treatment, comparator, care providers, centers, and blinding status.

1b-i) Key features/functionalities/components of the intervention and comparator in the METHODS section of the ABSTRACT

Mention key features/functionalities/components of the intervention and comparator in the abstract. If possible, also mention theories and principles used for designing the site. Keep in mind the needs of systematic reviewers and indexers by including important synonyms. (Note: Only report in the abstract what the main paper is reporting. If this information is missing from the main body of text, consider adding it)

	1	2	3	4	5	
subitem not at all important	○	○	○	○	◉	essential

Clear selection

You're editing your response. Sharing this URL allows others to also edit your response.

FILL IN A NEW RESPONSE

Does your paper address subitem 1b-i? *

Copy and paste relevant sections from the manuscript abstract (include quotes in quotation marks "like this" to indicate direct quotes from your manuscript), or elaborate on this item by providing additional information not in the ms, or briefly explain why the item is not applicable/relevant for your study

"Participants attended a synchronous 20-minute consultant surgeon-lead online lecture before 1:1 randomization (computer-generated sequence) to either a consultant surgeon-lead 10-minute metaverse-based VR session (3D Organon's Medverse on the PICO 4 Ultra headset) or a 10-minute 2D animated lecture."

1b-ii) Level of human involvement in the METHODS section of the ABSTRACT

Clarify the level of human involvement in the abstract, e.g., use phrases like “fully automated” vs. “therapist/nurse/care provider/physician-assisted” (mention number and expertise of providers involved, if any). (Note: Only report in the abstract what the main paper is reporting. If this information is missing from the main body of text, consider adding it)

	1	2	3	4	5	
subitem not at all important	○	○	○	○	◉	essential

Clear selection

Does your paper address subitem 1b-ii?

Copy and paste relevant sections from the manuscript abstract (include quotes in quotation marks "like this" to indicate direct quotes from your manuscript), or elaborate on this item by providing additional information not in the ms, or briefly explain why the item is not applicable/relevant for your study

"Participants attended a synchronous 20-minute consultant surgeon-lead online lecture before 1:1 randomization (computer-generated sequence) to either a consultant surgeon-lead 10-minute metaverse-based VR session (3D Organon's Medverse on the PICO 4 Ultra headset) or a 10-minute 2D animated lecture."

You're editing your response. Sharing this URL allows others to also edit your response.

FILL IN A NEW RESPONSE

1b-iii) Open vs. closed, web-based (self-assessment) vs. face-to-face assessments in the METHODS section of the ABSTRACT

Mention how participants were recruited (online vs. offline), e.g., from an open access website or from a clinic or a closed online user group (closed usergroup trial), and clarify if this was a purely web-based trial, or there were face-to-face components (as part of the intervention or for assessment). Clearly say if outcomes were self-assessed through questionnaires (as common in web-based trials). Note: In traditional offline trials, an open trial (open-label trial) is a type of clinical trial in which both the researchers and participants know which treatment is being administered. To avoid confusion, use “blinded” or “unblinded” to indicated the level of blinding instead of “open”, as “open” in web-based trials usually refers to “open access” (i.e. participants can self-enrol). (Note: Only report in the abstract what the main paper is reporting. If this information is missing from the main body of text, consider adding it)

	1	2	3	4	5	
subitem not at all important	○	○	○	○	◉	essential

Clear selection

Does your paper address subitem 1b-iii?

Copy and paste relevant sections from the manuscript abstract (include quotes in quotation marks "like this" to indicate direct quotes from your manuscript), or elaborate on this item by providing additional information not in the ms, or briefly explain why the item is not applicable/relevant for your study

"This prospective, randomized pilot study followed CONSORT-EHEALTH guidelines. Medical students from UK and Irish universities were recruited via convenience sampling through a surgical society network."

"Allocation was concealed until intervention; however, participants and researchers were unblinded. Data were collected via Google Forms at three intervals (baseline, post-intervention, and post-crossover) to assess confidence, spatial understanding, and knowledge (10-item MCQ)."

You're editing your response. Sharing this URL allows others to also edit your response.

FILL IN A NEW RESPONSE

1b-iv) RESULTS section in abstract must contain use data

Report number of participants enrolled/assessed in each group, the use/uptake of the intervention (e.g., attrition/adherence metrics, use over time, number of logins etc.), in addition to primary/secondary outcomes. (Note: Only report in the abstract what the main paper is reporting. If this information is missing from the main body of text, consider adding it)

	1	2	3	4	5	
subitem not at all important	○	○	○	○	◉	essential

Clear selection

Does your paper address subitem 1b-iv?

Copy and paste relevant sections from the manuscript abstract (include quotes in quotation marks "like this" to indicate direct quotes from your manuscript), or elaborate on this item by providing additional information not in the ms, or briefly explain why the item is not applicable/relevant for your study

"Twenty-four medical students from 11 UK and Irish medical schools participated; 92% reported no prior tracheostomy anatomy teaching. Adherence was 100%, with all participants completing their assigned 10-minute intervention and all assessment points. Anatomical confidence improved significantly in the VR group compared with animation (mean change 1.58 ± 1.00 vs 0.50 ± 0.80, P = 0.012). Knowledge scores improved significantly in both groups (VR: +1.75 ± 1.54, P = 0.007; Animation: +2.83 ± 1.70, P = 0.003), with no significant post-intervention difference between groups (p > 0.46). VR participants reported significantly superior spatial understanding across all measured domains (all P ≤ 0.009)."

You're editing your response. Sharing this URL allows others to also edit your response.

FILL IN A NEW RESPONSE

1b-v) CONCLUSIONS/DISCUSSION in abstract for negative trials

Conclusions/Discussions in abstract for negative trials: Discuss the primary outcome - if the trial is negative (primary outcome not changed), and the intervention was not used, discuss whether negative results are attributable to lack of uptake and discuss reasons. (Note: Only report in the abstract what the main paper is reporting. If this information is missing from the main body of text, consider adding it)

	1	2	3	4	5	
subitem not at all important	(•)	( )	( )	( )	( )	essential

Clear selection

Does your paper address subitem 1b-v?

Copy and paste relevant sections from the manuscript abstract (include quotes in quotation marks "like this" to indicate direct quotes from your manuscript), or elaborate on this item by providing additional information not in the ms, or briefly explain why the item is not applicable/relevant for your study

Not applicable. This is not a negative trial; the intervention showed significant between-group benefits for confidence and spatial understanding.

INTRODUCTION

2a) In INTRODUCTION: Scientific background and explanation of rationale

You're editing your response. Sharing this URL allows others to also edit your response.

FILL IN A NEW RESPONSE

2a-i) Problem and the type of system/solution

Describe the problem and the type of system/solution that is object of the study: intended as stand-alone intervention vs. incorporated in broader health care program? Intended for a particular patient population? Goals of the intervention, e.g., being more cost-effective to other interventions, replace or complement other solutions? (Note: Details about the intervention are provided in “Methods” under 5)

	1	2	3	4	5	
subitem not at all important	○	○	○	○	◉	essential

Clear selection

Does your paper address subitem 2a-i? *

Copy and paste relevant sections from the manuscript (include quotes in quotation marks "like this" to indicate direct quotes from your manuscript), or elaborate on this item by providing additional information not in the ms, or briefly explain why the item is not applicable/relevant for your study

The study addresses limited access to high-quality in-person anatomy education, particularly for tracheostomy anatomy, a topic where spatial understanding is critical for patient safety. Traditional anatomy teaching relies on cadaveric dissection and physical resources with restricted access. The intervention (3D Organon Medverse) is evaluated as a stand-alone educational session intended to complement traditional anatomy teaching. It is aimed at medical students (and the wider healthcare profession) who lack access to consistent cadaveric facilities. The goal is to assess whether synchronous remote VR is a scalable, effective alternative or adjunct to conventional 2D digital resources.

You're editing your response. Sharing this URL allows others to also edit your response.

FILL IN A NEW RESPONSE

2a-ii) Scientific background, rationale: What is known about the (type of) system

Scientific background, rationale: What is known about the (type of) system that is the object of the study (be sure to discuss the use of similar systems for other conditions/diagnoses, if appropiate), motivation for the study, i.e. what are the reasons for and what is the context for this specific study, from which stakeholder viewpoint is the study performed, potential impact of findings [2]. Briefly justify the choice of the comparator.

	1	2	3	4	5	
subitem not at all important	○	○	○	○	◉	essential

Clear selection

Does your paper address subitem 2a-ii? *

Copy and paste relevant sections from the manuscript (include quotes in quotation marks "like this" to indicate direct quotes from your manuscript), or elaborate on this item by providing additional information not in the ms, or briefly explain why the item is not applicable/relevant for your study

"Virtual reality (VR) provides an immersive digital learning environment capable of rendering detailed 3D anatomical models that students can explore remotely and interactively. By allowing learners to visualize structures from multiple perspectives and manipulate their spatial orientation, VR offers potential advantages over passive modalities. Recent research suggests that VR can improve knowledge acquisition, retention, learning confidence, and conceptual comprehension, making it an appealing adjunct or alternative to traditional anatomy teaching, especially in settings where access to cadaveric facilities is limited [2, 3]."

"Our primary objective was to evaluate whether VR enhances learner confidence and perceived spatial understanding of tracheostomy anatomy beyond that achievable with a conventional 2D resource. In addition, we examined changes in anatomical knowledge to determine whether immersive VR offers measurable educational benefits when delivered remotely within an undergraduate medical curriculum."

2b) In INTRODUCTION: Specific objectives or hypotheses

You're editing your response. Sharing this URL allows others to also edit your response.

FILL IN A NEW RESPONSE

Does your paper address CONSORT subitem 2b? *

Copy and paste relevant sections from the manuscript (include quotes in quotation marks "like this" to indicate direct quotes from your manuscript), or elaborate on this item by providing additional information not in the ms, or briefly explain why the item is not applicable/relevant for your study

"Our primary objective was to evaluate whether VR enhances learner confidence and perceived spatial understanding of tracheostomy anatomy beyond that achievable with a conventional 2D resource. In addition, we examined changes in anatomical knowledge to determine whether immersive VR offers measurable educational benefits when delivered remotely within an undergraduate medical curriculum."

METHODS

3a) Description of trial design (such as parallel, factorial) including allocation ratio

Does your paper address CONSORT subitem 3a? *

Copy and paste relevant sections from the manuscript (include quotes in quotation marks "like this" to indicate direct quotes from your manuscript), or elaborate on this item by providing additional information not in the ms, or briefly explain why the item is not applicable/relevant for your study

Yes. "This was a prospective randomized controlled pilot study". Participants were randomized in a 1:1 ratio using a computer-generated random sequence to receive either the VR session or the 2D animation first, then crossed over to the alternate modality. Primary between-group comparisons were made post-first-intervention.

3b) Important changes to methods after trial commencement (such as eligibility criteria), with reasons

You're editing your response. Sharing this URL allows others to also edit your response.

FILL IN A NEW RESPONSE

Does your paper address CONSORT subitem 3b? *

Copy and paste relevant sections from the manuscript (include quotes in quotation marks "like this" to indicate direct quotes from your manuscript), or elaborate on this item by providing additional information not in the ms, or briefly explain why the item is not applicable/relevant for your study

No important changes to methods were made after the trial commenced. The study was conducted as a single-session event on November 2, 2025; all procedures followed the pre-specified protocol.

3b-i) Bug fixes, Downtimes, Content Changes

Bug fixes, Downtimes, Content Changes: ehealth systems are often dynamic systems. A description of changes to methods therefore also includes important changes made on the intervention or comparator during the trial (e.g., major bug fixes or changes in the functionality or content) (5-iii) and other "unexpected events" that may have influenced study design such as staff changes, system failures/downtimes, etc. [2].

	1	2	3	4	5	
subitem not at all important	(●)	( )	( )	( )	( )	essential

Clear selection

Does your paper address subitem 3b-i?

Copy and paste relevant sections from the manuscript (include quotes in quotation marks "like this" to indicate direct quotes from your manuscript), or elaborate on this item by providing additional information not in the ms, or briefly explain why the item is not applicable/relevant for your study

No bug fixes, platform downtimes, or content changes occurred during the trial. The Medverse platform version 2025.2.6 was used throughout. No unexpected technical events affected study delivery.

4a) Eligibility criteria for participants

You're editing your response. Sharing this URL allows others to also edit your response. FILL IN A NEW RESPONSE

Does your paper address CONSORT subitem 4a? *

Copy and paste relevant sections from the manuscript (include quotes in quotation marks "like this" to indicate direct quotes from your manuscript), or elaborate on this item by providing additional information not in the ms, or briefly explain why the item is not applicable/relevant for your study

Yes. Inclusion criteria: current enrolment in a medical degree programme and access to Zoom. Exclusion criteria: inability to access necessary hardware (VR headset) or declining consent. Participants needed to register via a digital screening form verifying these criteria.

4a-i) Computer / Internet literacy

Computer / Internet literacy is often an implicit “de facto” eligibility criterion - this should be explicitly clarified.

	1	2	3	4	5	
subitem not at all important	○	○	◉	○	○	essential

Clear selection

Does your paper address subitem 4a-i?

Copy and paste relevant sections from the manuscript (include quotes in quotation marks "like this" to indicate direct quotes from your manuscript), or elaborate on this item by providing additional information not in the ms, or briefly explain why the item is not applicable/relevant for your study

Access to Zoom was an explicit eligibility criterion, implying basic internet literacy. VR headsets (PICO 4 Ultra) were distributed by regional leads before the study, removing the hardware access barrier. However, explicit assessment of computer literacy or prior VR experience was not an eligibility criterion. Prior VR experience was captured as a baseline variable; 83% had no prior synchronized VR experience.

You're editing your response. Sharing this URL allows others to also edit your response.

FILL IN A NEW RESPONSE

4a-ii) Open vs. closed, web-based vs. face-to-face assessments:

Open vs. closed, web-based vs. face-to-face assessments: Mention how participants were recruited (online vs. offline), e.g., from an open access website or from a clinic, and clarify if this was a purely web-based trial, or there were face-to-face components (as part of the intervention or for assessment), i.e., to what degree got the study team to know the participant. In online-only trials, clarify if participants were quasi-anonymous and whether having multiple identities was possible or whether technical or logistical measures (e.g., cookies, email confirmation, phone calls) were used to detect/prevent these.

	1	2	3	4	5	
subitem not at all important	○	○	○	○	◉	essential

Clear selection

Does your paper address subitem 4a-ii? *

Copy and paste relevant sections from the manuscript (include quotes in quotation marks "like this" to indicate direct quotes from your manuscript), or elaborate on this item by providing additional information not in the ms, or briefly explain why the item is not applicable/relevant for your study

Yes. This was an open online trial. Recruitment was conducted through the VRiMS national surgical society network and was open to the general public. The study was fully remote; there were no face-to-face components. All outcomes were self-assessed via Google Forms. Participant identity was quasi-anonymous: demographic data were limited to year of training and institutional affiliation to protect anonymity.

4a-iii) Information giving during recruitment

Information given during recruitment. Specify how participants were briefed for recruitment and in the informed consent procedures (e.g., publish the informed consent documentation as appendix, see also item X26), as this information may have an effect on user self-selection, user expectation and may also bias results.

	1	2	3	4	5	
subitem not at all important	○	○	○	◉	○	essential

Clear selection

You're editing your response. Sharing this URL allows others to also edit your response.

FILL IN A NEW RESPONSE

Does your paper address subitem 4a-iii?

Copy and paste relevant sections from the manuscript (include quotes in quotation marks "like this" to indicate direct quotes from your manuscript), or elaborate on this item by providing additional information not in the ms, or briefly explain why the item is not applicable/relevant for your study

Recruitment was conducted via multi-modal digital outreach (VRiMS social media, regional student leads). Interested students completed a digital screening form. Participants were provided a participant information sheet prior to providing informed consent. The consent process is described in the Ethical Considerations section. Providing VR headsets in advance may have increased expectations of the VR intervention.

4b) Settings and locations where the data were collected

Does your paper address CONSORT subitem 4b? *

Copy and paste relevant sections from the manuscript (include quotes in quotation marks "like this" to indicate direct quotes from your manuscript), or elaborate on this item by providing additional information not in the ms, or briefly explain why the item is not applicable/relevant for your study

Yes. The study was conducted remotely on November 2, 2025, with participants joining from their individual geographical locations across the UK and Republic of Ireland (11 universities, 7 regions) via Zoom and the Medverse platform.

4b-i) Report if outcomes were (self-)assessed through online questionnaires

Clearly report if outcomes were (self-)assessed through online questionnaires (as common in web-based trials) or otherwise.

	1	2	3	4	5	
subitem not at all important	○	○	○	○	◉	essential

Clear selection

You're editing your response. Sharing this URL allows others to also edit your response.

FILL IN A NEW RESPONSE

Does your paper address subitem 4b-i? *

Copy and paste relevant sections from the manuscript (include quotes in quotation marks "like this" to indicate direct quotes from your manuscript), or elaborate on this item by providing additional information not in the ms, or briefly explain why the item is not applicable/relevant for your study

Yes. All outcomes were self-assessed via three Google Forms questionnaires (Parts 1, 2, and 3), administered online at baseline (pre-lecture), post-intervention, and post-crossover respectively. Measures included 5-point Likert scales for confidence and spatial understanding, and a 10-item MCQ for knowledge.

4b-ii) Report how institutional affiliations are displayed

Report how institutional affiliations are displayed to potential participants [on ehealth media], as affiliations with prestigious hospitals or universities may affect volunteer rates, use, and reactions with regards to an intervention.(Not a required item – describe only if this may bias results)

	1	2	3	4	5	
subitem not at all important	○	◉	○	○	○	essential

Clear selection

Does your paper address subitem 4b-ii?

Copy and paste relevant sections from the manuscript (include quotes in quotation marks "like this" to indicate direct quotes from your manuscript), or elaborate on this item by providing additional information not in the ms, or briefly explain why the item is not applicable/relevant for your study

Not specifically reported. Recruitment was conducted through the VRiMS national network, which has university representatives in almost every UK and Irish medical schools. The consultant surgeon delivering the intervention was identifiable as a specialist. These associations may have influenced volunteer rates or participant expectations; this is acknowledged as a potential source of bias.

5) The interventions for each group with sufficient details to allow replication,

You're editing your response. Sharing this URL allows others to also edit your response.

FILL IN A NEW RESPONSE

5-i) Mention names, credential, affiliations of the developers, sponsors, and owners

Mention names, credential, affiliations of the developers, sponsors, and owners [6] (if authors/evaluators are owners or developer of the software, this needs to be declared in a "Conflict of interest" section or mentioned elsewhere in the manuscript).

	1	2	3	4	5	
subitem not at all important	○	◉	○	○	○	essential

Clear selection

Does your paper address subitem 5-i?

Copy and paste relevant sections from the manuscript (include quotes in quotation marks "like this" to indicate direct quotes from your manuscript), or elaborate on this item by providing additional information not in the ms, or briefly explain why the item is not applicable/relevant for your study

The intervention used 3D Organon's Medverse platform on the PICO 4 Ultra Enterprise headset (PICO Technology). Professor Dhanda is the CEO of Virtual Reality in Medicine and Surgery (VRiMS), and is part of the Scientific Advisory Board of 3D Organon and served as the academic supervisor for this project. He was not involved in data collection or statistical analysis. This is declared in the Conflict of Interest section. He was not involved in data collection or statistical analysis.

5-ii) Describe the history/development process

Describe the history/development process of the application and previous formative evaluations (e.g., focus groups, usability testing), as these will have an impact on adoption/use rates and help with interpreting results.

	1	2	3	4	5	
subitem not at all important	◉	○	○	○	○	essential

Clear selection

You're editing your response. Sharing this URL allows others to also edit your response.

FILL IN A NEW RESPONSE

Does your paper address subitem 5-ii?

Copy and paste relevant sections from the manuscript (include quotes in quotation marks "like this" to indicate direct quotes from your manuscript), or elaborate on this item by providing additional information not in the ms, or briefly explain why the item is not applicable/relevant for your study

Not described. Medverse is a commercially available metaverse network module from 3D Organon; its prior development or formative evaluation is not discussed in the manuscript. No formative usability testing specific to this study population was conducted before the trial.

5-iii) Revisions and updating

Revisions and updating. Clearly mention the date and/or version number of the application/intervention (and comparator, if applicable) evaluated, or describe whether the intervention underwent major changes during the evaluation process, or whether the development and/or content was “frozen” during the trial. Describe dynamic components such as news feeds or changing content which may have an impact on the replicability of the intervention (for unexpected events see item 3b).

	1	2	3	4	5	
subitem not at all important	○	○	○	◉	○	essential

Clear selection

Does your paper address subitem 5-iii?

Copy and paste relevant sections from the manuscript (include quotes in quotation marks "like this" to indicate direct quotes from your manuscript), or elaborate on this item by providing additional information not in the ms, or briefly explain why the item is not applicable/relevant for your study

The version of 3D Organon Medverse used was 2025.2.6. The study was conducted on a single day (November 2, 2025); no changes to the platform content were made during the trial. The platform content was effectively 'frozen' for the duration of the session.

You're editing your response. Sharing this URL allows others to also edit your response.

FILL IN A NEW RESPONSE

5-iv) Quality assurance methods

Provide information on quality assurance methods to ensure accuracy and quality of information provided [1], if applicable.

	1	2	3	4	5	
subitem not at all important	○	○	○	○	◉	essential

Clear selection

Does your paper address subitem 5-iv?

Copy and paste relevant sections from the manuscript (include quotes in quotation marks "like this" to indicate direct quotes from your manuscript), or elaborate on this item by providing additional information not in the ms, or briefly explain why the item is not applicable/relevant for your study

Yes. The VR content was delivered live by a consultant surgeon following four predefined learning objectives derived from the National Tracheostomy Safety Project e-learning module, developed by NHS England and the Royal College of Anaesthetists. The comparator video was also aligned to these four objectives and was narrated by an associate professor in head and neck surgery.

5-v) Ensure replicability by publishing the source code, and/or providing screenshots/screen-capture video, and/or providing flowcharts of the algorithms used

Ensure replicability by publishing the source code, and/or providing screenshots/screen-capture video, and/or providing flowcharts of the algorithms used. Replicability (i.e., other researchers should in principle be able to replicate the study) is a hallmark of scientific reporting.

	1	2	3	4	5	
subitem not at all important	○	○	○	◉	○	essential

Clear selection

You're editing your response. Sharing this URL allows others to also edit your response.

FILL IN A NEW RESPONSE

Does your paper address subitem 5-v?

Copy and paste relevant sections from the manuscript (include quotes in quotation marks "like this" to indicate direct quotes from your manuscript), or elaborate on this item by providing additional information not in the ms, or briefly explain why the item is not applicable/relevant for your study

A screenshot of the Medverse platform (Figure 2) is included, showing participants in the virtual room. The CONSORT flow diagram (Figure 1) illustrates the study structure. All three questionnaires are provided in Appendices 1–4. The YouTube comparator video is a publicly accessible resource. Source code for the VR platform is proprietary to 3D Organon.

5-vi) Digital preservation

Digital preservation: Provide the URL of the application, but as the intervention is likely to change or disappear over the course of the years; also make sure the intervention is archived (Internet Archive, webcitation.org, and/or publishing the source code or screenshots/videos alongside the article). As pages behind login screens cannot be archived, consider creating demo pages which are accessible without login.

	1	2	3	4	5	
subitem not at all important	○	○	○	○	◉	essential

Clear selection

Does your paper address subitem 5-vi?

Copy and paste relevant sections from the manuscript (include quotes in quotation marks "like this" to indicate direct quotes from your manuscript), or elaborate on this item by providing additional information not in the ms, or briefly explain why the item is not applicable/relevant for your study

The 3D Organon website URL (https://www.3dorganon.com/) is provided. The YouTube comparator video reference [8] is cited. No formal archiving (e.g., Internet Archive, WebCite) of the intervention was performed, as the platform requires login access.

You're editing your response. Sharing this URL allows others to also edit your response.

FILL IN A NEW RESPONSE

5-vii) Access

Access: Describe how participants accessed the application, in what setting/context, if they had to pay (or were paid) or not, whether they had to be a member of specific group. If known, describe how participants obtained “access to the platform and Internet” [1]. To ensure access for editors/reviewers/readers, consider to provide a “backdoor” login account or demo mode for reviewers/readers to explore the application (also important for archiving purposes, see vi).

	1	2	3	4	5	
subitem not at all important	○	○	○	○	◉	essential

Clear selection

Does your paper address subitem 5-vii? *

Copy and paste relevant sections from the manuscript (include quotes in quotation marks "like this" to indicate direct quotes from your manuscript), or elaborate on this item by providing additional information not in the ms, or briefly explain why the item is not applicable/relevant for your study

Yes. All participants were provided PICO 4 Ultra headsets by VRiMS regional leads before the study, at no cost. Access to the Medverse VR session was via the headsets during a Zoom-coordinated synchronous session. The control group accessed the YouTube animation via their own internet-connected devices. There was no cost to participants. Access was provided within the closed study session only.

You're editing your response. Sharing this URL allows others to also edit your response.

FILL IN A NEW RESPONSE

5-viii) Mode of delivery, features/functionalities/components of the intervention and comparator, and the theoretical framework

Describe mode of delivery, features/functionalities/components of the intervention and comparator, and the theoretical framework [6] used to design them (instructional strategy [1], behaviour change techniques, persuasive features, etc., see e.g., [7, 8] for terminology). This includes an in-depth description of the content (including where it is coming from and who developed it) [1]," whether [and how] it is tailored to individual circumstances and allows users to track their progress and receive feedback" [6]. This also includes a description of communication delivery channels and – if computer-mediated communication is a component – whether communication was synchronous or asynchronous [6]. It also includes information on presentation strategies [1], including page design principles, average amount of text on pages, presence of hyperlinks to other resources, etc. [1].

	1	2	3	4	5	
subitem not at all important	○	○	○	○	◉	essential

Clear selection

Does your paper address subitem 5-viii? *

Copy and paste relevant sections from the manuscript (include quotes in quotation marks "like this" to indicate direct quotes from your manuscript), or elaborate on this item by providing additional information not in the ms, or briefly explain why the item is not applicable/relevant for your study

Yes. Intervention: 10-minute synchronous VR session in Medverse (3DOrganon) on PICO 4 Ultra (6DoF headset); live consultant surgeon demonstrated tracheostomy anatomy including rotation and spatial navigation of cervical structures with real-time verbal explanation. Comparator: 10-minute pre-recorded 2D YouTube animation with verbal narration, viewed passively. Both modalities were matched to four learning objectives. Theoretical framework: Cognitive Affective Model of Immersive Learning (CAMIL), supporting questionnaire design targeting self-efficacy, presence, engagement, and knowledge outcomes.

You're editing your response. Sharing this URL allows others to also edit your response.

FILL IN A NEW RESPONSE

5-ix) Describe use parameters

Describe use parameters (e.g., intended “doses” and optimal timing for use). Clarify what instructions or recommendations were given to the user, e.g., regarding timing, frequency, heaviness of use, if any, or was the intervention used ad libitum.

	1	2	3	4	5	
subitem not at all important	○	○	○	○	◉	essential

Clear selection

Does your paper address subitem 5-ix?

Copy and paste relevant sections from the manuscript (include quotes in quotation marks "like this" to indicate direct quotes from your manuscript), or elaborate on this item by providing additional information not in the ms, or briefly explain why the item is not applicable/relevant for your study

Yes. Each modality was a single 10-minute session, delivered once within a structured 60-minute workshop. Participants were not instructed to use the platform independently outside the study session. The intervention was administered as a fixed, instructor-led dose with no self-directed or repeated use component.

5-x) Clarify the level of human involvement

Clarify the level of human involvement (care providers or health professionals, also technical assistance) in the e-intervention or as co-intervention (detail number and expertise of professionals involved, if any, as well as “type of assistance offered, the timing and frequency of the support, how it is initiated, and the medium by which the assistance is delivered”. It may be necessary to distinguish between the level of human involvement required for the trial, and the level of human involvement required for a routine application outside of a RCT setting (discuss under item 21 – generalizability).

	1	2	3	4	5	
subitem not at all important	○	○	○	○	◉	essential

Clear selection

You're editing your response. Sharing this URL allows others to also edit your response.

FILL IN A NEW RESPONSE

Does your paper address subitem 5-x?

Copy and paste relevant sections from the manuscript (include quotes in quotation marks "like this" to indicate direct quotes from your manuscript), or elaborate on this item by providing additional information not in the ms, or briefly explain why the item is not applicable/relevant for your study

Yes. VR arm: consultant surgeon delivered real-time live instruction, demonstrating anatomy with verbal explanations, including spatial navigation. Control arm: passive viewing of a pre-recorded animation; no live human involvement. This asymmetry is identified as a potential confound and limitation in the manuscript.

5-xi) Report any prompts/reminders used

Report any prompts/reminders used: Clarify if there were prompts (letters, emails, phone calls, SMS) to use the application, what triggered them, frequency etc. It may be necessary to distinguish between the level of prompts/reminders required for the trial, and the level of prompts/reminders for a routine application outside of a RCT setting (discuss under item 21 – generalizability).

	1	2	3	4	5	
subitem not at all important	○	○	○	○	◉	essential

Clear selection

Does your paper address subitem 5-xi? *

Copy and paste relevant sections from the manuscript (include quotes in quotation marks "like this" to indicate direct quotes from your manuscript), or elaborate on this item by providing additional information not in the ms, or briefly explain why the item is not applicable/relevant for your study

"To minimize non-response bias, automated reminders were sent 24 hours. Of the 4 students who initially registered and were sent hardware, were unable to attend due to technical or personal conflicts, resulting in a final analytical sample of n=24. There was 0% attrition (drop-out) once the session commenced, as all participants completed the final questionnaire."

You're editing your response. Sharing this URL allows others to also edit your response.

FILL IN A NEW RESPONSE

5-xii) Describe any co-interventions (incl. training/support)

Describe any co-interventions (incl. training/support): Clearly state any interventions that are provided in addition to the targeted eHealth intervention, as ehealth intervention may not be designed as stand-alone intervention. This includes training sessions and support [1]. It may be necessary to distinguish between the level of training required for the trial, and the level of training for a routine application outside of a RCT setting (discuss under item 21 – generalizability.

	1	2	3	4	5	
subitem not at all important	○	○	○	◉	○	essential

Clear selection

Does your paper address subitem 5-xii? *

Copy and paste relevant sections from the manuscript (include quotes in quotation marks "like this" to indicate direct quotes from your manuscript), or elaborate on this item by providing additional information not in the ms, or briefly explain why the item is not applicable/relevant for your study

Yes. A key co-intervention common to both arms was a 20-minute synchronous didactic lecture delivered by the same consultant surgeon prior to randomization, covering the four tracheostomy learning objectives. VR headsets were distributed and set up before the study date, with no formal VR training session. In a routine application setting, formal headset orientation training may be required.

6a) Completely defined pre-specified primary and secondary outcome measures, including how and when they were assessed

You're editing your response. Sharing this URL allows others to also edit your response.

FILL IN A NEW RESPONSE

Does your paper address CONSORT subitem 6a? *

Copy and paste relevant sections from the manuscript (include quotes in quotation marks "like this" to indicate direct quotes from your manuscript), or elaborate on this item by providing additional information not in the ms, or briefly explain why the item is not applicable/relevant for your study

Yes. Primary outcomes: (1) self-reported anatomical confidence assessed pre- and post-intervention via 5-point Likert scale; (2) anatomical knowledge via 10-item best-of-five MCQ (Parts 1 and 2). Secondary outcomes: spatial understanding and perspective-taking via 5-point Likert scale (Part 2 only); telepresence (Temple Presence Inventory adapted items, Part 3); modality preference (Part 3). All outcomes collected via Google Forms immediately after each assessment point.

6a-i) Online questionnaires: describe if they were validated for online use and apply CHERRIES items to describe how the questionnaires were designed/deployed

If outcomes were obtained through online questionnaires, describe if they were validated for online use and apply CHERRIES items to describe how the questionnaires were designed/deployed [9].

	1	2	3	4	5	
subitem not at all important	○	○	◉	○	○	essential

Clear selection

Does your paper address subitem 6a-i?

Copy and paste relevant sections from manuscript text

The questionnaires were not formally validated prior to deployment. They were informed by the Cognitive Affective Model of Immersive Learning (CAMIL) and adapted from the Temple Presence Inventory (a validated instrument). This limitation is acknowledged in the manuscript. Questionnaires were delivered as self-administered, untimed Google Forms.

You're editing your response. Sharing this URL allows others to also edit your response.

FILL IN A NEW RESPONSE

6a-ii) Describe whether and how “use” (including intensity of use/dosage) was defined/measured/monitored

Describe whether and how “use” (including intensity of use/dosage) was defined/measured/monitored (logins, logfile analysis, etc.). Use/adoption metrics are important process outcomes that should be reported in any ehealth trial.

	1	2	3	4	5	
subitem not at all important	○	○	○	○	◉	essential

Clear selection

Does your paper address subitem 6a-ii?

Copy and paste relevant sections from manuscript text

Yes. Use was defined as completion of the assigned 10-minute modality. Adherence was monitored as a binary outcome (completion/non-completion) and reported as 100%. No log-file or login data were collected from the platform. Session duration was fixed and synchronized for all participants.

6a-iii) Describe whether, how, and when qualitative feedback from participants was obtained

Describe whether, how, and when qualitative feedback from participants was obtained (e.g., through emails, feedback forms, interviews, focus groups).

	1	2	3	4	5	
subitem not at all important	○	○	○	◉	○	essential

Clear selection

You're editing your response. Sharing this URL allows others to also edit your response.

FILL IN A NEW RESPONSE

Does your paper address subitem 6a-iii?

Copy and paste relevant sections from manuscript text

Part 3 of the questionnaire included comparative questions asking participants to indicate which modality they found more engaging and which had greater perceived educational impact. These were structured Likert items, not open-ended qualitative feedback. No separate interviews or focus groups were conducted.

6b) Any changes to trial outcomes after the trial commenced, with reasons

Does your paper address CONSORT subitem 6b? *

Copy and paste relevant sections from the manuscript (include quotes in quotation marks "like this" to indicate direct quotes from your manuscript), or elaborate on this item by providing additional information not in the ms, or briefly explain why the item is not applicable/relevant for your study

No changes to trial outcomes were made after the trial commenced. All outcomes were pre-specified prior to the study date.

7a) How sample size was determined

NPT: When applicable, details of whether and how the clustering by care provides or centers was addressed

7a-i) Describe whether and how expected attrition was taken into account when calculating the sample size

Describe whether and how expected attrition was taken into account when calculating the sample size.

	1	2	3	4	5	
subitem not at all important	○	○	○	○	●	essential

Clear selection

You're editing your response. Sharing this URL allows others to also edit your response.

FILL IN A NEW RESPONSE

Does your paper address subitem 7a-i?

Copy and paste relevant sections from manuscript title (include quotes in quotation marks "like this" to indicate direct quotes from your manuscript), or elaborate on this item by providing additional information not in the ms, or briefly explain why the item is not applicable/relevant for your study

Yes. No formal power calculation was performed. Target sample size was n=24 (12 per arm), following Julious (2005) recommendation for pilot randomized controlled trials, considered sufficient to provide preliminary estimates of mean and variance for primary outcomes to power a future definitive trial.

7b) When applicable, explanation of any interim analyses and stopping guidelines

Does your paper address CONSORT subitem 7b? *

Copy and paste relevant sections from the manuscript (include quotes in quotation marks "like this" to indicate direct quotes from your manuscript), or elaborate on this item by providing additional information not in the ms, or briefly explain why the item is not applicable/relevant for your study

Not applicable. Given the single-session pilot design and small sample size, no interim analyses or stopping guidelines were planned or implemented.

8a) Method used to generate the random allocation sequence

NPT: When applicable, how care providers were allocated to each trial group

Does your paper address CONSORT subitem 8a? *

Copy and paste relevant sections from the manuscript (include quotes in quotation marks "like this" to indicate direct quotes from your manuscript), or elaborate on this item by providing additional information not in the ms, or briefly explain why the item is not applicable/relevant for your study

Yes. A computer-generated random sequence was used to allocate participants in a 1:1 ratio.

You're editing your response. Sharing this URL allows others to also edit your response.

FILL IN A NEW RESPONSE

8b) Type of randomisation; details of any restriction (such as blocking and block size)

Does your paper address CONSORT subitem 8b? *

Copy and paste relevant sections from the manuscript (include quotes in quotation marks "like this" to indicate direct quotes from your manuscript), or elaborate on this item by providing additional information not in the ms, or briefly explain why the item is not applicable/relevant for your study

Simple 1:1 randomization via computer-generated sequence. No blocking or stratification was applied. This is described as a potential source of imbalance given the small sample size (n=24), but groups were comparable on all measured baseline characteristics (Table 1).

9) Mechanism used to implement the random allocation sequence (such as sequentially numbered containers), describing any steps taken to conceal the sequence until interventions were assigned

Does your paper address CONSORT subitem 9? *

Copy and paste relevant sections from the manuscript (include quotes in quotation marks "like this" to indicate direct quotes from your manuscript), or elaborate on this item by providing additional information not in the ms, or briefly explain why the item is not applicable/relevant for your study

Yes. 'Allocation was concealed until the commencement of the intervention phase.' Participants were not informed of their group assignment until after the shared didactic lecture, immediately prior to starting their assigned intervention.

10) Who generated the random allocation sequence, who enrolled participants, and who assigned participants to interventions

You're editing your response. Sharing this URL allows others to also edit your response.

FILL IN A NEW RESPONSE

Does your paper address CONSORT subitem 10? *

Copy and paste relevant sections from the manuscript (include quotes in quotation marks "like this" to indicate direct quotes from your manuscript), or elaborate on this item by providing additional information not in the ms, or briefly explain why the item is not applicable/relevant for your study

Partially. The joint first authros (JH and AG) generated the random allocation sequence, enrolled participants and assigned participants to interventions. They are listed as responsible for project administration in the author contributions section.

11a) If done, who was blinded after assignment to interventions (for example, participants, care providers, those assessing outcomes) and how

NPT: Whether or not administering co-interventions were blinded to group assignment

11a-i) Specify who was blinded, and who wasn't

Specify who was blinded, and who wasn't. Usually, in web-based trials it is not possible to blind the participants [1, 3] (this should be clearly acknowledged), but it may be possible to blind outcome assessors, those doing data analysis or those administering co-interventions (if any).

	1	2	3	4	5	
subitem not at all important	○	○	○	○	◉	essential

Clear selection

Does your paper address subitem 11a-i? *

Copy and paste relevant sections from the manuscript (include quotes in quotation marks "like this" to indicate direct quotes from your manuscript), or elaborate on this item by providing additional information not in the ms, or briefly explain why the item is not applicable/relevant for your study

Yes. 'Due to the nature of the interventions, neither participants nor researchers were blinded to group allocation.' Blinding was not possible given the overt difference between VR and 2D animation. Outcome assessors were also unblinded as assessments were self-reported. This is acknowledged as a limitation.

You're editing your response. Sharing this URL allows others to also edit your response.

FILL IN A NEW RESPONSE

11a-ii) Discuss e.g., whether participants knew which intervention was the "intervention of interest" and which one was the "comparator"

Informed consent procedures (4a-ii) can create biases and certain expectations - discuss e.g., whether participants knew which intervention was the "intervention of interest" and which one was the "comparator".

	1	2	3	4	5	
subitem not at all important	○	○	○	◉	○	essential

Clear selection

Does your paper address subitem 11a-ii?

Copy and paste relevant sections from the manuscript (include quotes in quotation marks "like this" to indicate direct quotes from your manuscript), or elaborate on this item by providing additional information not in the ms, or briefly explain why the item is not applicable/relevant for your study

Yes, implicitly. Given recruitment through a VR-focused society (VRiMS), participants were likely aware that VR was the intervention under evaluation. This may have introduced performance bias, particularly for subjective outcomes (confidence, spatial understanding). This is acknowledged in the limitations via the discussion of self-selection bias and 'novelty effect.'

11b) If relevant, description of the similarity of interventions

(this item is usually not relevant for ehealth trials as it refers to similarity of a placebo or sham intervention to a active medication/intervention)

Does your paper address CONSORT subitem 11b? *

Copy and paste relevant sections from the manuscript (include quotes in quotation marks "like this" to indicate direct quotes from your manuscript), or elaborate on this item by providing additional information not in the ms, or briefly explain why the item is not applicable/relevant for your study

Not applicable. There was no placebo or sham intervention. Both arms received active educational content (VR vs. 2D animation), matched on topic, duration, and learning [illegible]

You're editing your response. Sharing this URL allows others to also edit your response.

FILL IN A NEW RESPONSE

12a) Statistical methods used to compare groups for primary and secondary outcomes

NPT: When applicable, details of whether and how the clustering by care providers or centers was addressed

Does your paper address CONSORT subitem 12a? *

Copy and paste relevant sections from the manuscript (include quotes in quotation marks "like this" to indicate direct quotes from your manuscript), or elaborate on this item by providing additional information not in the ms, or briefly explain why the item is not applicable/relevant for your study

Yes. Normality assessed via Shapiro-Wilk test. As data were not normally distributed, non-parametric tests were applied: Wilcoxon signed-rank test (within-group paired comparisons); Mann-Whitney U test (between-group comparisons); Chi-square or Fisher's exact test (categorical variables). Likert data summarized as mean ± SD. Statistical significance: P < 0.05. Analysis conducted in SPSS v31.

12a-i) Imputation techniques to deal with attrition / missing values

Imputation techniques to deal with attrition / missing values: Not all participants will use the intervention/comparator as intended and attrition is typically high in ehealth trials. Specify how participants who did not use the application or dropped out from the trial were treated in the statistical analysis (a complete case analysis is strongly discouraged, and simple imputation techniques such as LOCF may also be problematic [4]).

	1	2	3	4	5	
subitem not at all important	◉	○	○	○	○	essential

Clear selection

You're editing your response. Sharing this URL allows others to also edit your response.

FILL IN A NEW RESPONSE

Does your paper address subitem 12a-i? *

Copy and paste relevant sections from the manuscript (include quotes in quotation marks "like this" to indicate direct quotes from your manuscript), or elaborate on this item by providing additional information not in the ms, or briefly explain why the item is not applicable/relevant for your study

No missing data were identified; therefore, no imputation was required. All 24 participants completed all three questionnaire time points. The 0% attrition rate means a complete case analysis is equivalent to an intent-to-treat analysis for this trial.

12b) Methods for additional analyses, such as subgroup analyses and adjusted analyses

Does your paper address CONSORT subitem 12b? *

Copy and paste relevant sections from the manuscript (include quotes in quotation marks "like this" to indicate direct quotes from your manuscript), or elaborate on this item by providing additional information not in the ms, or briefly explain why the item is not applicable/relevant for your study

Yes. Post-crossover analyses (Part 3) assessed comparative participant preferences and perceptions of both modalities. Telepresence and agency following VR were explored descriptively. These were pre-specified secondary analyses.

X26) REB/IRB Approval and Ethical Considerations [recommended as subheading under "Methods"] (not a CONSORT item)

X26-i) Comment on ethics committee approval

	1	2	3	4	5	
subitem not at all important	○	○	○	○	◉	essential

Clear selection

You're editing your response. Sharing this URL allows others to also edit your response.

FILL IN A NEW RESPONSE

Does your paper address subitem X26-i?

Copy and paste relevant sections from the manuscript (include quotes in quotation marks "like this" to indicate direct quotes from your manuscript), or elaborate on this item by providing additional information not in the ms, or briefly explain why the item is not applicable/relevant for your study

Yes. 'Ethical approval for this study was granted by the Brighton and Sussex Medical School (BSMS) Research Governance and Ethics Committee (RGEC), reference number ER/BSMS9GYI/1, in accordance with the Declaration of Helsinki and UK GDPR regulations.'

x26-ii) Outline informed consent procedures

Outline informed consent procedures e.g., if consent was obtained offline or online (how? Checkbox, etc.?), and what information was provided (see 4a-ii). See [6] for some items to be included in informed consent documents.

	1	2	3	4	5	
subitem not at all important	○	○	○	○	◉	essential

Clear selection

Does your paper address subitem X26-ii?

Copy and paste relevant sections from the manuscript (include quotes in quotation marks "like this" to indicate direct quotes from your manuscript), or elaborate on this item by providing additional information not in the ms, or briefly explain why the item is not applicable/relevant for your study

Yes. 'Participation was voluntary with participants providing informed consent, after reading the participant information sheet, and agreement, for their data to be used towards this research study.' Consent was obtained online prior to study commencement. The participant information sheet was distributed digitally.

You're editing your response. Sharing this URL allows others to also edit your response.

FILL IN A NEW RESPONSE

X26-iii) Safety and security procedures

Safety and security procedures, incl. privacy considerations, and any steps taken to reduce the likelihood or detection of harm (e.g., education and training, availability of a hotline)

	1	2	3	4	5	
subitem not at all important	○	○	○	○	◉	essential

Clear selection

Does your paper address subitem X26-iii?

Copy and paste relevant sections from the manuscript (include quotes in quotation marks "like this" to indicate direct quotes from your manuscript), or elaborate on this item by providing additional information not in the ms, or briefly explain why the item is not applicable/relevant for your study

Yes. Data collection was conducted in compliance with UK GDPR regulations. Participant anonymity was prioritized: demographic data were limited to year of training and institutional affiliation only (no age, gender, or ethnicity collected). No adverse events or privacy breaches occurred during the trial.

RESULTS

13a) For each group, the numbers of participants who were randomly assigned, received intended treatment, and were analysed for the primary outcome

NPT: The number of care providers or centers performing the intervention in each group and the number of patients treated by each care provider in each center

You're editing your response. Sharing this URL allows others to also edit your response.

FILL IN A NEW RESPONSE

Does your paper address CONSORT subitem 13a? *

Copy and paste relevant sections from the manuscript (include quotes in quotation marks "like this" to indicate direct quotes from your manuscript), or elaborate on this item by providing additional information not in the ms, or briefly explain why the item is not applicable/relevant for your study

Yes. 28 students were initially recruited; 4 were excluded prior to participation (3 did not meet inclusion criteria, 1 declined consent). 24 participants were randomized (VR n=12, Control n=12). All 24 were analyzed for primary outcomes. 100% completed both modalities and all assessment time points.

13b) For each group, losses and exclusions after randomisation, together with reasons

Does your paper address CONSORT subitem 13b? (NOTE: Preferably, this is shown in a CONSORT flow diagram) *

Copy and paste relevant sections from the manuscript (include quotes in quotation marks "like this" to indicate direct quotes from your manuscript), or elaborate on this item by providing additional information not in the ms, or briefly explain why the item is not applicable/relevant for your study

Yes. A CONSORT 2025 flow diagram (Figure 1) is included. No participants were lost to follow-up or withdrew post-randomization. Four exclusions occurred prior to randomization: 3 ineligible, 1 declined consent.

13b-i) Attrition diagram

Strongly recommended: An attrition diagram (e.g., proportion of participants still logging in or using the intervention/comparator in each group plotted over time, similar to a survival curve) or other figures or tables demonstrating usage/dose/engagement.

	1	2	3	4	5	
subitem not at all important	(●)	( )	( )	( )	( )	essential

Clear selection

You're editing your response. Sharing this URL allows others to also edit your response.

FILL IN A NEW RESPONSE

Does your paper address subitem 13b-i?

Copy and paste relevant sections from the manuscript or cite the figure number if applicable (include quotes in quotation marks "like this" to indicate direct quotes from your manuscript), or elaborate on this item by providing additional information not in the ms, or briefly explain why the item is not applicable/relevant for your study

A CONSORT flow diagram is presented (Figure 1). A separate attrition/usage diagram was not produced as adherence was 100% throughout the single-session study. There was no differential dropout or usage to display over time.

14a) Dates defining the periods of recruitment and follow-up

Does your paper address CONSORT subitem 14a? *

Copy and paste relevant sections from the manuscript (include quotes in quotation marks "like this" to indicate direct quotes from your manuscript), or elaborate on this item by providing additional information not in the ms, or briefly explain why the item is not applicable/relevant for your study

Yes. Recruitment began 10 days prior to the study date via VRiMS network outreach. The study was conducted on November 2, 2025. There was no longitudinal follow-up beyond the single 60-minute session.

14a-i) Indicate if critical “secular events” fell into the study period

Indicate if critical “secular events” fell into the study period, e.g., significant changes in Internet resources available or “changes in computer hardware or Internet delivery resources”

	1	2	3	4	5	
subitem not at all important	◉	○	○	○	○	essential

Clear selection

You're editing your response. Sharing this URL allows others to also edit your response.

FILL IN A NEW RESPONSE

Does your paper address subitem 14a-i?

Copy and paste relevant sections from the manuscript (include quotes in quotation marks "like this" to indicate direct quotes from your manuscript), or elaborate on this item by providing additional information not in the ms, or briefly explain why the item is not applicable/relevant for your study

No significant secular events (e.g., major changes to VR hardware, internet infrastructure, or curriculum) fell within the study period. The study was conducted as a single session, limiting exposure to such events.

14b) Why the trial ended or was stopped (early)

Does your paper address CONSORT subitem 14b? *

Copy and paste relevant sections from the manuscript (include quotes in quotation marks "like this" to indicate direct quotes from your manuscript), or elaborate on this item by providing additional information not in the ms, or briefly explain why the item is not applicable/relevant for your study

The trial was completed as planned. Target sample size (n=24) was achieved, all pre-specified assessments were completed, and there were no adverse events or reasons to stop the study early.

15) A table showing baseline demographic and clinical characteristics for each group

NPT: When applicable, a description of care providers (case volume, qualification, expertise, etc.) and centers (volume) in each group

Does your paper address CONSORT subitem 15? *

Copy and paste relevant sections from the manuscript (include quotes in quotation marks "like this" to indicate direct quotes from your manuscript), or elaborate on this item by providing additional information not in the ms, or briefly explain why the item is not applicable/relevant for your study

Yes. Table 1 presents baseline characteristics by group including: year of medical training, regional affiliation, prior tracheostomy teaching, prior VR experience, and Likert-scored

You're editing your response. Sharing this URL allows others to also edit your response.

FILL IN A NEW RESPONSE

15-i) Report demographics associated with digital divide issues

In ehealth trials it is particularly important to report demographics associated with digital divide issues, such as age, education, gender, social-economic status, computer/Internet/ehealth literacy of the participants, if known.

	1	2	3	4	5	
subitem not at all important	○	○	◉	○	○	essential

Clear selection

Does your paper address subitem 15-i? *

Copy and paste relevant sections from the manuscript (include quotes in quotation marks "like this" to indicate direct quotes from your manuscript), or elaborate on this item by providing additional information not in the ms, or briefly explain why the item is not applicable/relevant for your study

Partially. Age, gender, race/ethnicity, and socioeconomic status were not collected, to protect participant anonymity and streamline remote recruitment. This is a limitation acknowledged in the manuscript. Prior VR experience (a proxy for digital literacy) was captured; 83% had no prior synchronized VR experience. Participants were all medical students with internet access and ability to use Zoom, implying baseline digital literacy.

16) For each group, number of participants (denominator) included in each analysis and whether the analysis was by original assigned groups

16-i) Report multiple “denominators” and provide definitions

Report multiple “denominators” and provide definitions: Report N’s (and effect sizes) “across a range of study participation [and use] thresholds” [1], e.g., N exposed, N consented, N used more than x times, N used more than y weeks, N participants “used” the intervention/comparator at specific pre-defined time points of interest (in absolute and relative numbers per group). Always clearly define “use” of the intervention.

	1	2	3	4	5	
subitem not at all important	○	○	○	○	◉	essential

You're editing your response. Sharing this URL allows others to also edit your response.

FILL IN A NEW RESPONSE

Does your paper address subitem 16-i? *

Copy and paste relevant sections from the manuscript (include quotes in quotation marks "like this" to indicate direct quotes from your manuscript), or elaborate on this item by providing additional information not in the ms, or briefly explain why the item is not applicable/relevant for your study

Yes. All 24 randomized participants were analyzed by original assigned group (n=12 VR, n=12 Control). No participants were excluded from analysis. Denominators reported: N initially recruited = 28; N excluded pre-randomization = 4; N randomized = 24 (12 per arm); N completing all assessments = 24. 'Use' was defined as completion of the assigned 10-minute modality. All 24 participants met this threshold (100% adherence).

16-ii) Primary analysis should be intent-to-treat

Primary analysis should be intent-to-treat, secondary analyses could include comparing only “users”, with the appropriate caveats that this is no longer a randomized sample (see 18-i).

	1	2	3	4	5	
subitem not at all important	○	○	○	○	◉	essential

Clear selection

Does your paper address subitem 16-ii?

Copy and paste relevant sections from the manuscript (include quotes in quotation marks "like this" to indicate direct quotes from your manuscript), or elaborate on this item by providing additional information not in the ms, or briefly explain why the item is not applicable/relevant for your study

Yes, effectively. Given 0% post-randomization attrition, all randomized participants were included in all analyses. The complete case analysis is equivalent to intent-to-treat in this context, as there were no missing outcomes.

17a) For each primary and secondary outcome, results for each group, and the estimated effect size and its precision (such as 95% confidence interval)

You're editing your response. Sharing this URL allows others to also edit your response.

FILL IN A NEW RESPONSE

Does your paper address CONSORT subitem 17a? *

Copy and paste relevant sections from the manuscript (include quotes in quotation marks "like this" to indicate direct quotes from your manuscript), or elaborate on this item by providing additional information not in the ms, or briefly explain why the item is not applicable/relevant for your study

Yes. Confidence: VR mean change +1.58 ± 1.00 vs control +0.50 ± 0.80 (P=0.012 between-group). Knowledge: VR +1.75 ± 1.54 (P=0.007 within), control +2.83 ± 1.70 (P=0.003 within); no between-group difference (P>0.46). Spatial understanding: VR significantly superior on all four domains (all P ≤ 0.009). Results presented as mean ± SD with P values. Confidence intervals were not reported; this is a limitation.

17a-i) Presentation of process outcomes such as metrics of use and intensity of use

In addition to primary/secondary (clinical) outcomes, the presentation of process outcomes such as metrics of use and intensity of use (dose, exposure) and their operational definitions is critical. This does not only refer to metrics of attrition (13-b) (often a binary variable), but also to more continuous exposure metrics such as "average session length". These must be accompanied by a technical description how a metric like a "session" is defined (e.g., timeout after idle time) [1] (report under item 6a).

	1	2	3	4	5	
subitem not at all important	○	○	○	○	◉	essential

Clear selection

Does your paper address subitem 17a-i?

Copy and paste relevant sections from the manuscript (include quotes in quotation marks "like this" to indicate direct quotes from your manuscript), or elaborate on this item by providing additional information not in the ms, or briefly explain why the item is not applicable/relevant for your study

Yes. Adherence was 100%: all 24 participants completed their assigned 10-minute intervention. Session duration was fixed at 10 minutes per modality. No platform-generated usage logs were available; adherence was operationally defined as completion of the post-intervention questionnaire (Part 2).

You're editing your response. Sharing this URL allows others to also edit your response.

FILL IN A NEW RESPONSE

17b) For binary outcomes, presentation of both absolute and relative effect sizes is recommended

Does your paper address CONSORT subitem 17b? *

Copy and paste relevant sections from the manuscript (include quotes in quotation marks "like this" to indicate direct quotes from your manuscript), or elaborate on this item by providing additional information not in the ms, or briefly explain why the item is not applicable/relevant for your study

Not applicable. Primary outcomes were continuous (Likert scale) and ordinal (MCQ score); no binary outcomes were analyzed. Percentage change values were reported alongside mean differences for interpretability (e.g., +79% for VR confidence, +20% for control confidence).

18) Results of any other analyses performed, including subgroup analyses and adjusted analyses, distinguishing pre-specified from exploratory

Does your paper address CONSORT subitem 18? *

Copy and paste relevant sections from the manuscript (include quotes in quotation marks "like this" to indicate direct quotes from your manuscript), or elaborate on this item by providing additional information not in the ms, or briefly explain why the item is not applicable/relevant for your study

Yes. Post-crossover data (Part 3) were analyzed to assess comparative modality preference, telepresence, and perceived agency following VR. These were pre-specified secondary analyses. No post-hoc subgroup analyses were performed.

You're editing your response. Sharing this URL allows others to also edit your response.

FILL IN A NEW RESPONSE

18-i) Subgroup analysis of comparing only users

A subgroup analysis of comparing only users is not uncommon in ehealth trials, but if done, it must be stressed that this is a self-selected sample and no longer an unbiased sample from a randomized trial (see 16-iii).

	1	2	3	4	5	
subitem not at all important	(●)	( )	( )	( )	( )	essential

Clear selection

Does your paper address subitem 18-i?

Copy and paste relevant sections from the manuscript (include quotes in quotation marks "like this" to indicate direct quotes from your manuscript), or elaborate on this item by providing additional information not in the ms, or briefly explain why the item is not applicable/relevant for your study

Not applicable. All randomized participants completed the intervention (100% adherence). There was no subset of 'users vs non-users' to distinguish; no differential analysis by use was needed.

19) All important harms or unintended effects in each group

(for specific guidance see CONSORT for harms)

Does your paper address CONSORT subitem 19? *

Copy and paste relevant sections from the manuscript (include quotes in quotation marks "like this" to indicate direct quotes from your manuscript), or elaborate on this item by providing additional information not in the ms, or briefly explain why the item is not applicable/relevant for your study

Yes. No adverse events, harms, or unintended negative effects were reported. No participants experienced discomfort, technical injury, or cybersickness requiring withdrawal.

You're editing your response. Sharing this URL allows others to also edit your response. FILL IN A NEW RESPONSE

19-i) Include privacy breaches, technical problems

Include privacy breaches, technical problems. This does not only include physical “harm” to participants, but also incidents such as perceived or real privacy breaches [1], technical problems, and other unexpected/unintended incidents. “Unintended effects” also includes unintended positive effects [2].

	1	2	3	4	5	
subitem not at all important	○	○	○	◉	○	essential

Clear selection

Does your paper address subitem 19-i?

Copy and paste relevant sections from the manuscript (include quotes in quotation marks "like this" to indicate direct quotes from your manuscript), or elaborate on this item by providing additional information not in the ms, or briefly explain why the item is not applicable/relevant for your study

No privacy breaches occurred. No significant technical problems were reported during the session. Hardware was distributed and tested in advance. The study was conducted fully in compliance with UK GDPR regulations.

19-ii) Include qualitative feedback from participants or observations from staff/researchers

Include qualitative feedback from participants or observations from staff/researchers, if available, on strengths and shortcomings of the application, especially if they point to unintended/unexpected effects or uses. This includes (if available) reasons for why people did or did not use the application as intended by the developers.

	1	2	3	4	5	
subitem not at all important	○	○	○	○	◉	essential

Clear selection

You're editing your response. Sharing this URL allows others to also edit your response.

FILL IN A NEW RESPONSE

Does your paper address subitem 19-ii?

Copy and paste relevant sections from the manuscript (include quotes in quotation marks "like this" to indicate direct quotes from your manuscript), or elaborate on this item by providing additional information not in the ms, or briefly explain why the item is not applicable/relevant for your study

Partially. Part 3 of the questionnaire captured comparative participant preferences between modalities, including which was more engaging and which had greater perceived educational impact. VR was rated significantly higher on engagement and perceived educational impact. No open-ended qualitative feedback mechanism was included. Researcher observations were not formally recorded.

DISCUSSION

22) Interpretation consistent with results, balancing benefits and harms, and considering other relevant evidence

NPT: In addition, take into account the choice of the comparator, lack of or partial blinding, and unequal expertise of care providers or centers in each group

22-i) Restate study questions and summarize the answers suggested by the data, starting with primary outcomes and process outcomes (use)

Restate study questions and summarize the answers suggested by the data, starting with primary outcomes and process outcomes (use).

	1	2	3	4	5	
subitem not at all important	○	○	○	○	◉	essential

Clear selection

You're editing your response. Sharing this URL allows others to also edit your response.

FILL IN A NEW RESPONSE

Does your paper address subitem 22-i? *

Copy and paste relevant sections from the manuscript (include quotes in quotation marks "like this" to indicate direct quotes from your manuscript), or elaborate on this item by providing additional information not in the ms, or briefly explain why the item is not applicable/relevant for your study

Yes. The discussion restates the primary aims and summarizes findings: VR was non-inferior for factual knowledge but significantly superior for confidence and spatial understanding. The conclusion states: 'While it appears non-inferior to standard animations for factual recall, it is significantly superior for developing spatial understanding and learner confidence.'

22-ii) Highlight unanswered new questions, suggest future research

Highlight unanswered new questions, suggest future research.

	1	2	3	4	5	
subitem not at all important	○	○	○	○	◉	essential

Clear selection

Does your paper address subitem 22-ii?

Copy and paste relevant sections from the manuscript (include quotes in quotation marks "like this" to indicate direct quotes from your manuscript), or elaborate on this item by providing additional information not in the ms, or briefly explain why the item is not applicable/relevant for your study

Yes. Future directions include: (1) larger cohorts with adequate power; (2) longitudinal follow-up to assess knowledge retention; (3) investigation of VR scalability to resource-limited institutions; (4) controlled comparison of synchronous instructor-led VR vs. unguided VR to isolate the effect of immersive modality from instructor presence.

20) Trial limitations, addressing sources of potential bias, imprecision, and, if relevant, multiplicity of analyses

You're editing your response. Sharing this URL allows others to also edit your response.

FILL IN A NEW RESPONSE

20-i) Typical limitations in ehealth trials

Typical limitations in ehealth trials: Participants in ehealth trials are rarely blinded. Ehealth trials often look at a multiplicity of outcomes, increasing risk for a Type I error. Discuss biases due to non-use of the intervention/usability issues, biases through informed consent procedures, unexpected events.

	1	2	3	4	5	
subitem not at all important	○	○	○	○	◉	essential

Clear selection

Does your paper address subitem 20-i? *

Copy and paste relevant sections from the manuscript (include quotes in quotation marks "like this" to indicate direct quotes from your manuscript), or elaborate on this item by providing additional information not in the ms, or briefly explain why the item is not applicable/relevant for your study

Yes. Key limitations discussed: (1) small sample (n=24), limiting statistical power and generalizability; (2) self-selection bias — participants recruited through VR-focused society with high baseline interest in VR (mean 4.9/5); (3) confounding co-intervention: VR arm received live instructor, control arm viewed pre-recorded video; (4) unblinded design due to nature of interventions; (5) test-retest effect on knowledge MCQ; (6) subjective Likert scales susceptible to novelty effect; (7) 5-point ceiling effect on confidence scores; (8) hardware access and cost as barriers to scalability.

21) Generalisability (external validity, applicability) of the trial findings

NPT: External validity of the trial findings according to the intervention, comparators, patients, and care providers or centers involved in the trial

You're editing your response. Sharing this URL allows others to also edit your response.

FILL IN A NEW RESPONSE

21-i) Generalizability to other populations

Generalizability to other populations: In particular, discuss generalizability to a general Internet population, outside of a RCT setting, and general patient population, including applicability of the study results for other organizations

	1	2	3	4	5	
subitem not at all important	○	○	○	○	◉	essential

Clear selection

Does your paper address subitem 21-i?

Copy and paste relevant sections from the manuscript (include quotes in quotation marks "like this" to indicate direct quotes from your manuscript), or elaborate on this item by providing additional information not in the ms, or briefly explain why the item is not applicable/relevant for your study

Yes. The discussion addresses generalizability: VR delivered successfully to students across 11 UK/Irish medical schools demonstrates scalability. However, the self-selected, VR-enthusiastic sample limits generalizability to the broader medical student population. The paper discusses applicability to resource-limited settings and institutions with limited cadaver access. Generalizability outside an RCT setting (without provided headsets, structured session, and live instructor) is limited.

21-ii) Discuss if there were elements in the RCT that would be different in a routine application setting

Discuss if there were elements in the RCT that would be different in a routine application setting (e.g., prompts/reminders, more human involvement, training sessions or other co-interventions) and what impact the omission of these elements could have on use, adoption, or outcomes if the intervention is applied outside of a RCT setting.

	1	2	3	4	5	
subitem not at all important	○	○	○	○	◉	essential

Clear selection

You're editing your response. Sharing this URL allows others to also edit your response.

FILL IN A NEW RESPONSE

Does your paper address subitem 21-ii?

Copy and paste relevant sections from the manuscript (include quotes in quotation marks "like this" to indicate direct quotes from your manuscript), or elaborate on this item by providing additional information not in the ms, or briefly explain why the item is not applicable/relevant for your study

Yes. Key differences discussed: (1) VR headsets were provided to all participants in the RCT; in routine settings, hardware acquisition would be required; (2) the RCT used a live consultant surgeon for VR delivery; routine application may use recorded or less specialized instructors; (3) the structured 60-minute session with reminders and researcher oversight is unlikely to be replicated outside an RCT.

OTHER INFORMATION

23) Registration number and name of trial registry

Does your paper address CONSORT subitem 23? *

Copy and paste relevant sections from the manuscript (include quotes in quotation marks "like this" to indicate direct quotes from your manuscript), or elaborate on this item by providing additional information not in the ms, or briefly explain why the item is not applicable/relevant for your study

We have not registered our trial as this is a pilot RCT.

24) Where the full trial protocol can be accessed, if available

Does your paper address CONSORT subitem 24? *

Cite a Multimedia Appendix, other reference, or copy and paste relevant sections from the manuscript (include quotes in quotation marks "like this" to indicate direct quotes from your manuscript), or elaborate on this item by providing additional information not in the ms, or briefly explain why the item is not applicable/relevant for your study

[illegible]

You're editing your response. Sharing this URL allows others to also edit your response.

FILL IN A NEW RESPONSE

25) Sources of funding and other support (such as supply of drugs), role of funders

Does your paper address CONSORT subitem 25? *

Copy and paste relevant sections from the manuscript (include quotes in quotation marks "like this" to indicate direct quotes from your manuscript), or elaborate on this item by providing additional information not in the ms, or briefly explain why the item is not applicable/relevant for your study

Yes. 'This research was supported by the ASiT Surgical Research Training Grant 2024. The funding body had no role in the design, data collection, analysis, interpretation, or writing of the manuscript.'

X27) Conflicts of Interest (not a CONSORT item)

X27-i) State the relation of the study team towards the system being evaluated

In addition to the usual declaration of interests (financial or otherwise), also state the relation of the study team towards the system being evaluated, i.e., state if the authors/evaluators are distinct from or identical with the developers/sponsors of the intervention.

	1	2	3	4	5	
subitem not at all important	○	○	○	○	◉	essential

Clear selection

You're editing your response. Sharing this URL allows others to also edit your response.

FILL IN A NEW RESPONSE

Does your paper address subitem X27-i?

Copy and paste relevant sections from the manuscript (include quotes in quotation marks "like this" to indicate direct quotes from your manuscript), or elaborate on this item by providing additional information not in the ms, or briefly explain why the item is not applicable/relevant for your study

Yes. 'Professor Dhanda is the CEO of Virtual Reality in Medicine and Surgery (VRiMS) and is a part of the Scientific Advisory Board of 3D Organon, and served as the academic supervisor for this project. He was not involved in data collection or statistical analysis. All other authors declare no conflicts of interest.' The evaluating authors (Jason Ha, Andrew Gan, and co-authors) are distinct from the developers of the 3D Organon/Medverse platform. There is no financial relationship between the study team and 3DOrganon.

About the CONSORT EHEALTH checklist

As a result of using this checklist, did you make changes in your manuscript? *

- ○ yes, major changes
- ◉ yes, minor changes
- ○ no

What were the most important changes you made as a result of using this checklist?

Very thorough breakdown of the CONSORT guidelines and allowed us to report every item.

How much time did you spend on going through the checklist INCLUDING making changes in your manuscript *

Over 5 hours editing and making changes.

You're editing your response. Sharing this URL allows others to also edit your response.

FILL IN A NEW RESPONSE

As a result of using this checklist, do you think your manuscript has improved? *

- (x) yes
- ( ) no
- ( ) Other:

Would you like to become involved in the CONSORT EHEALTH group?

This would involve for example becoming involved in participating in a workshop and writing an "Explanation and Elaboration" document

- ( ) yes
- (x) no
- ( ) Other:

Clear selection

Any other comments or questions on CONSORT EHEALTH

Your answer

STOP - Save this form as PDF before you click submit

To generate a record that you filled in this form, we recommend to generate a PDF of this page (on a Mac, simply select "print" and then select "print as PDF") before you submit it.

When you submit your (revised) paper to JMIR, please upload the PDF as supplementary file.

Don't worry if some text in the textboxes is cut off, as we still have the complete information in our database. Thank you!

Final step: Click submit !

You're editing your response. Sharing this URL allows others to also edit your response.

FILL IN A NEW RESPONSE

Submit

Never submit passwords through Google Forms.

This content is neither created nor endorsed by Google. - Contact form owner - Terms of Service - Privacy Policy

Does this form look suspicious? Report

Google Forms

You're editing your response. Sharing this URL allows others to also edit your response.

FILL IN A NEW RESPONSE

You're editing your response. Sharing this URL allows others to also edit your response.

FILL IN A NEW RESPONSE
